# Divergent processing strategies for auditory sequence recognition in humans and zebra finches (*Taeniopygia guttata*)

**DOI:** 10.1038/s42003-026-11035-9

**Published:** 2026-10-13

**Authors:** Zhi-Yuan Ning, Jiaxin Li, Carel ten Cate, Henkjan Honing

**Affiliations:** 1 Institute of Biology Leiden, Leiden University, Leiden, The Netherlands; 2 Music Cognition Group, Institute for Logic, Language and Computation, University of Amsterdam, Amsterdam, The Netherlands

**Keywords:** Human behaviour, Sensory processing, Signal processing

## Abstract

Deriving sequential information from complex sounds is a challenge for auditory systems, yet it remains unclear whether humans and nonhuman species integrate multidimensional cues or prioritize a single dimension when processing multidimensional input. We investigated how humans and zebra finches (*Taeniopygia guttata*) process concurrent acoustic cues using an identical behavioural paradigm with complex-tone sequences. By presenting probe stimuli that selectively preserved or disrupted either pitch or formant trajectory, we quantified these processing strategies. We show that zebra finches employ an additive integration strategy, treating pitch and formant trajectories as complementary components of a unified auditory pattern. In contrast, humans exhibit a dominant reliance on pitch. Experience further modulates this bias, with trained human listeners recruiting secondary cues to preserve learned relational structure when pitch is unreliable. These findings imply a cross-species divergence in auditory representation, suggesting that auditory processing is shaped by an interplay of species-specific priorities and experience-dependent functional substitution.

## Introduction

Music and speech share core organizational principles: both comprise systematically ordered sequences of acoustic events whose structure is supported by perceptual dimensions such as rhythm, pitch, and timbre^1^. Recent neuroimaging work has sharpened the spectrotemporal distinctions between these domains and revealed hemispheric asymmetries in sensitivity to spectral versus temporal modulation^2–4^. Although subcortical circuits may provide a common substrate for encoding basic acoustic dimensions across domains, higher-level cognitive processing diverges with functional demands. For instance, in speech, two key dimensions—pitch and formant—carry communicative and linguistic information (e.g., prosody and vowel identity), whereas in music these same acoustic dimensions function as abstract fundamental frequency- and timbre-based structures that support melodic and timbral organization^2,5,6^.

Importantly, sensitivity to pitch- and formant-related structure is not uniquely human: both dimensions contribute to vocal communication across diverse animal taxa^7–9^. Moreover, growing evidence indicates that perceptual categories such as “pitch” and “timbre” do not map transparently across species, motivating renewed attention to the underlying spectral percepts that listeners actually exploit^10^. The central unresolved issue is whether flexible integration or segregation of concurrent acoustic dimensions—depending on context and task demands—reflects a general property of auditory processing or, instead, a specialization shaped in humans by extensive experience and cultural exposure. Addressing this question demands comparative experiments that directly assess how humans and other vocal learners^11^, including songbirds, prioritize and combine pitch and formant contours, particularly when these cues vary in their congruence.

Pitch is conventionally defined as the perceptual correlate of a sound’s fundamental frequency (*f*₀)^12^. However, human listeners can also track spectral regularities and melodic contour in ways that do not require an explicit estimate of *f*₀^13,14^. For harmonic sounds, component frequencies are typically integrated into a single perceptual object^15^, and “melodic” structure can be supported by contours in other dimensions, including loudness and brightness^13^. Even with these alternative pathways, pitch remains a cornerstone of human music cognition and a primary substrate for organizing complex acoustic structure^16,17^. Across species, sensitivity to pitch varies markedly. Birds, for instance, often exceed many mammals on absolute-pitch discrimination^18–20^. Yet comparative work indicates that listeners in many taxa preferentially exploit other cues, such as spectral shape, rhythm, or overall sound patterning, rather than pitch per se (e.g., Hoeschele^9^, also see ten Cate & Honing^5^ for a more elaborate discussion). Consistent with this view, studies in rodents^21^ and songbirds^22,23^ suggest that pitch may not be perceptually dominant relative to various other spectrotemporal cues, or that its relevance can be stimulus-dependent^24^. Nevertheless, songbirds can perceive missing fundamentals^25^, or use pitch when it provides the most informative cue^26^.

In humans, formants—peaks in the sound spectrum (*spectral envelope*)—are a principal perceptual dimension and provide primary cues for vowel identity^27^. Sensitivity to formant structure is not uniquely human: a wide range of mammals^28–31^ and birds^32–36^ detect and exploit formant-related information in acoustic signals, supporting the view that spectral cues are broadly available for communication and auditory patterning across taxa.

However, interactions between perceptual dimensions may influence grouping and judgment, with congruent cue changes strengthening perceptual organization and incongruent changes weakening it^37–41^. In humans, the magnitude and direction of these interactions depend on task demands and listener experience^42,43^. In contrast, comparatively little is known about how such cue integration operates in nonhuman species, in part because most studies rely on acoustically sparse stimuli. Here, we test zebra finches and humans with sequences of harmonic complex tones in which pitch and formant contours vary either congruently (parallel contours) or incongruently (crossed contours).

The primary objective of this study was to assess how zebra finches and humans weigh co-occurring (whether congruent or incongruent) patterns of acoustic dimensions when discriminating sequences of complex tones. Specifically, we asked whether listeners preferentially rely on pitch or on formant contours or, instead, integrate both cues during sequence processing. We further tested whether cue weighting depends on the relationship between the two contours, that is, whether pitch and formant trajectories shift in the same direction (parallel, yielding a coherent composite pattern) or in opposite directions (potentially forcing selective attention to a single dimension). For humans, we examined whether cue weighting was affected by musical experience. We found a cross-species divergence in how harmonic sequence structure is encoded: from additive multi-cues integration in zebra finches to pitch-dominant reliance in humans, highlighting how species-typical auditory priorities and experience shape sequential sound representations.

## Results

### Cue weighting differs across species

**Fig. 1:**
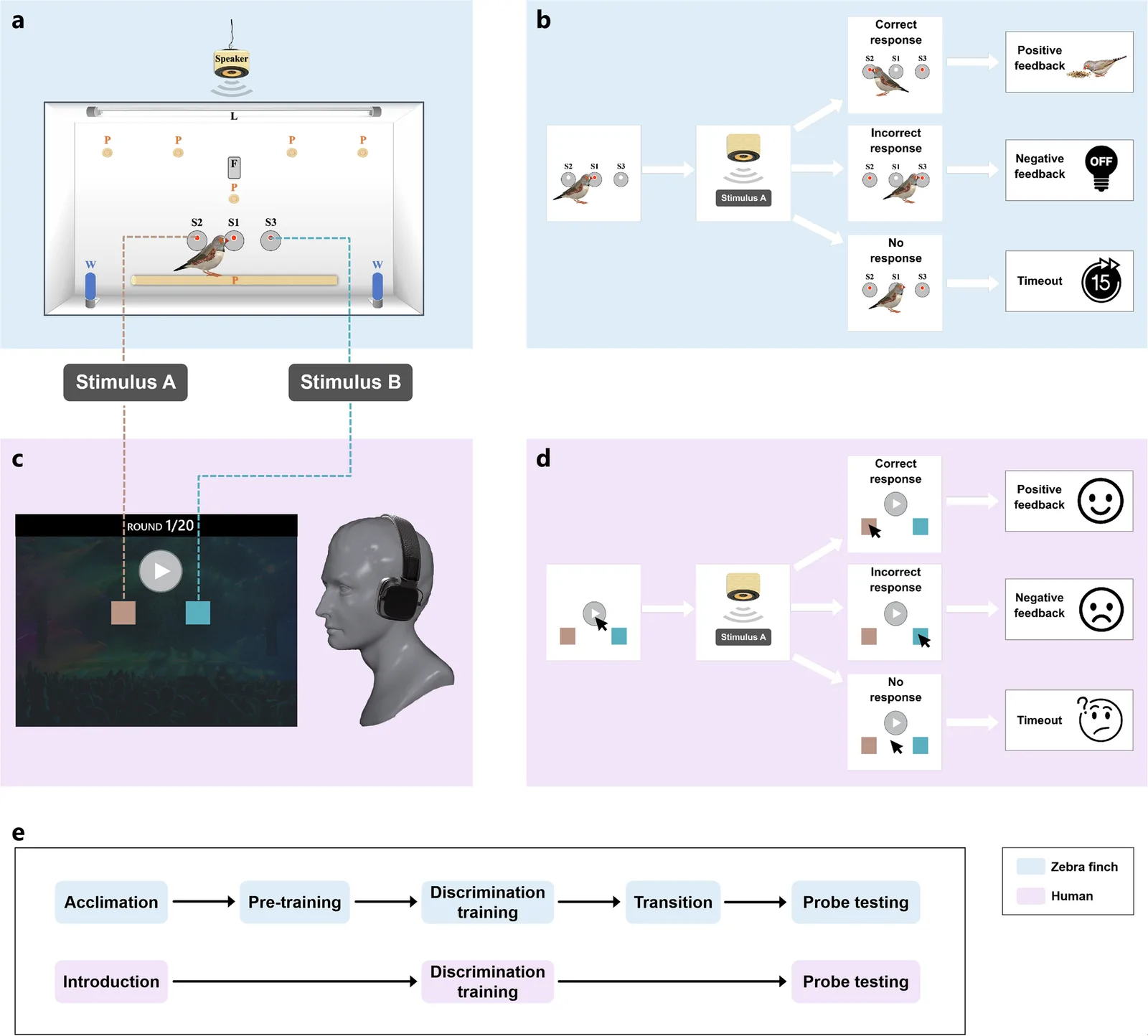
Apparatus and experimental procedure. **a** Schematic front view of the operant conditioning apparatus (Skinner box) used for the zebra finch experiment. A speaker (top of **a**) is suspended from the ceiling above the cage. Within the cage, there are several perches (P) for the bird to sit on. A food hatch (F) is located in the upper middle of the back panel, and a lamp (L) is placed at the top of the cage. Two tubes with ad libitum water (W) are placed symmetrically on two sides of the cage, and three sensors (S1, S2, S3) with red LEDs are lined horizontally in the lower middle of the back panel. **b** Example of a discrimination training trial in the zebra finch experiment. During the discrimination training phase, only the S1 LED was illuminated at the start of each trial. The bird first had to peck the center sensor (S1) to trigger the playback of a stimulus, and then choose between the two side sensors (S2 or S3), depending on the playback sound. A correct response—pecking the sensor associated with the presented stimulus (e.g., pecking S2 after stimulus A)—was rewarded with 12 s of food access. An incorrect response (e.g., pecking S3 after stimulus A) resulted in a 1-s light-off penalty. If the bird did not respond within 15 s, the trial ended automatically (Timeout) without a ‘positive’- or ‘negative’-feedback. **c** Schematic of the online listening categorization task for human participants (the “digital Skinner box”), analogous to the operant discrimination paradigm used in the zebra finch experiment. At the start of each trial, the computer screen displays a full-screen interface with a playback icon in the upper middle, which participants must click to initiate the playback of a sound sequence. Following the sound playback, two response buttons appear: one brown and one azure. The position of these buttons is fixed—placed next to each other below the playback button—though the specific assignment of brown and azure is randomized across participants. Participants must respond by clicking one of the buttons based on the sound they hear. **d** An example of the discrimination training trials in human online task. Mirroring the procedure in the zebra finch experiment, during the discrimination training phase, human participants have to learn to associate the playback sound with one of the two buttons: A correct response was followed by a smiley face as positive feedback, an incorrect response triggered a sad face as negative feedback, and no response within 3 s resulted in a Timeout (indicated by a confused face), after which the trial ended automatically. **e** Schematic of the experimental procedures for the zebra finch experiment and the human online task. Zebra finches completed acclimation/pre-training, discrimination training, a transition phase with reduced reinforcement, and a probe testing phase. Humans completed an introduction phase, discrimination training with feedback, and a probe testing phase. In both species, responses to probe stimuli were not scored for accuracy but were used to calculate similarity scores that quantified how the probe stimuli were classified relative to the training stimuli. See Methods for details.

**Fig. 2:**
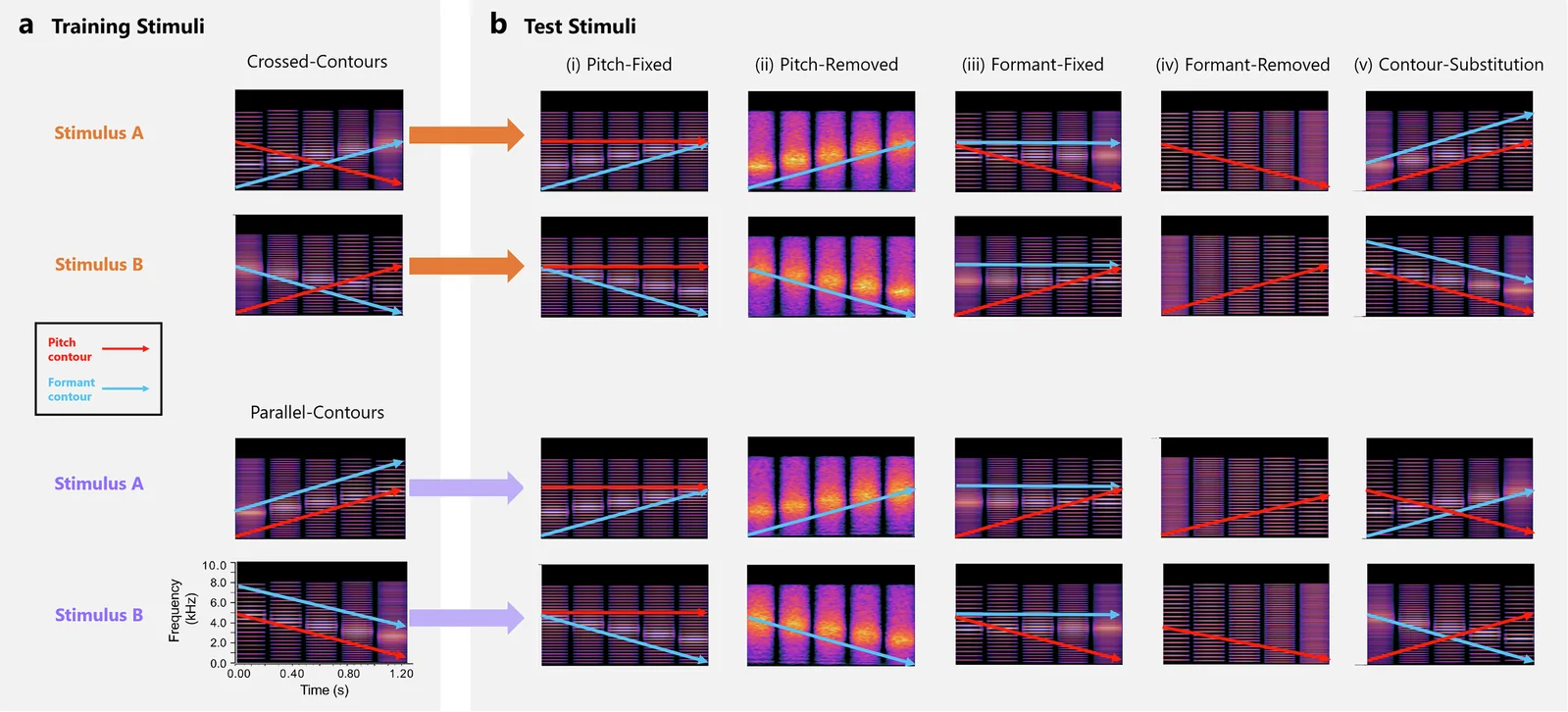
Illustration of stimuli used for songbird and human experiments. **a** Examples of a pair of training stimuli for the Crossed-Contours group (upper panel) and for the Parallel-Contours group (lower panel). **b** Modified stimuli used in the testing phase for the Crossed-Contours group (upper panel) and for the Parallel-Contours group (lower panel). Birds and human participants were tested with five modified versions of each training stimulus after completing the training: two pitch-modified versions (*pitch-fixed* and *pitch-removed*), two formant-modified versions (*formant-removed* and *formant-fixed*), and a *contour-substitution* version, which combined the formant contour of one training stimulus with the pitch contour of the other training stimulus — see Methods for a description of these manipulations. In the spectrograms of each tone sequence, the direction (but not the exact value) of the pitch contour is indicated by a red line, and formant contour is indicated by a blue line. All stimuli are accessible on the Open Science Framework (OSF) (DOI: 10.17605/OSF.IO/ZXDBJ).

We investigated the processing of pitch and formant information by testing both humans and zebra finches in a cross-species operant framework (Go-left/Go-right discrimination task), implemented via a physical apparatus (Skinner box) for birds and a digitally rendered “Skinner box” for humans, to ensure that differences in classification strategies reflected divergent auditory architectures rather than differences in task instruction or motivation (Fig. 1). During the training phase, subjects first learned to associate each of two training tone sequences (Stimulus A and Stimulus B) with one of two response options (left/right sensors for zebra finches; blue/brown buttons for humans) through trial-by-trial feedback (Fig. 1). Once the discrimination criterion had been reached, subjects proceeded to the testing phase, in which modified probe stimuli were presented to determine which acoustic cues guided their classification. Subjects heard five-tone training sequences in which pitch and formant contours either shifted in the same direction or in opposite directions (Fig. 2), a stimulus design that targeted sensitivity to the global contour of the sequence rather than to acoustic features of single tones. Hereafter probe stimuli examined whether the trained discrimination was based on having learned about the pattern of pitch, formant or both. This approach enabled us to quantify the relative weighting of pitch and formant cues under controlled manipulations of contour congruence. The results reveal striking differences between species as well as consistent variation within humans. Zebra finches relied on both pitch and formant cues in an integrated, additive fashion across all conditions, whereas humans exhibited a strong bias toward pitch. When pitch cues of probe stimuli were uninformative, musically experienced listeners showed enhanced flexibility in their use of spectral information.

### Zebra finches integrate pitch and formant

The responses to each probe stimulus were used to calculate a similarity score reflecting how closely the probe stimulus was classified as resembling either of the two training stimuli (A or B). Values near 1 reflect a strong association with its corresponding training stimulus (e.g., probe A with training A). Scores near 0.5 indicate ambiguous similarity, whereas a value near 0 means that a probe stimulus was considered similar to the opposite training stimulus (B). We also computed response rates, which indicate the proportion of trials on which subjects committed to a classification rather than withholding a response, and thus provide a complementary measure of perceptual confidence and stimulus classifiability, allowing us to dissociate cue-based biases (reduced similarity scores) from perceptual uncertainty (reduced responding). Additionally, we computed LogRatio to quantify response bias: values around zero represent the discrimination boundaries, where the probability of classifying a probe stimulus as similar to its corresponding training stimulus equals the probability of classifying it as similar to the opposite training stimulus, while positive or negative values indicate a significant bias for the probe stimulus associated with the corresponding or opposite training stimulus, respectively (see Methods for details).

#### Similarity scores

**Fig. 3:**
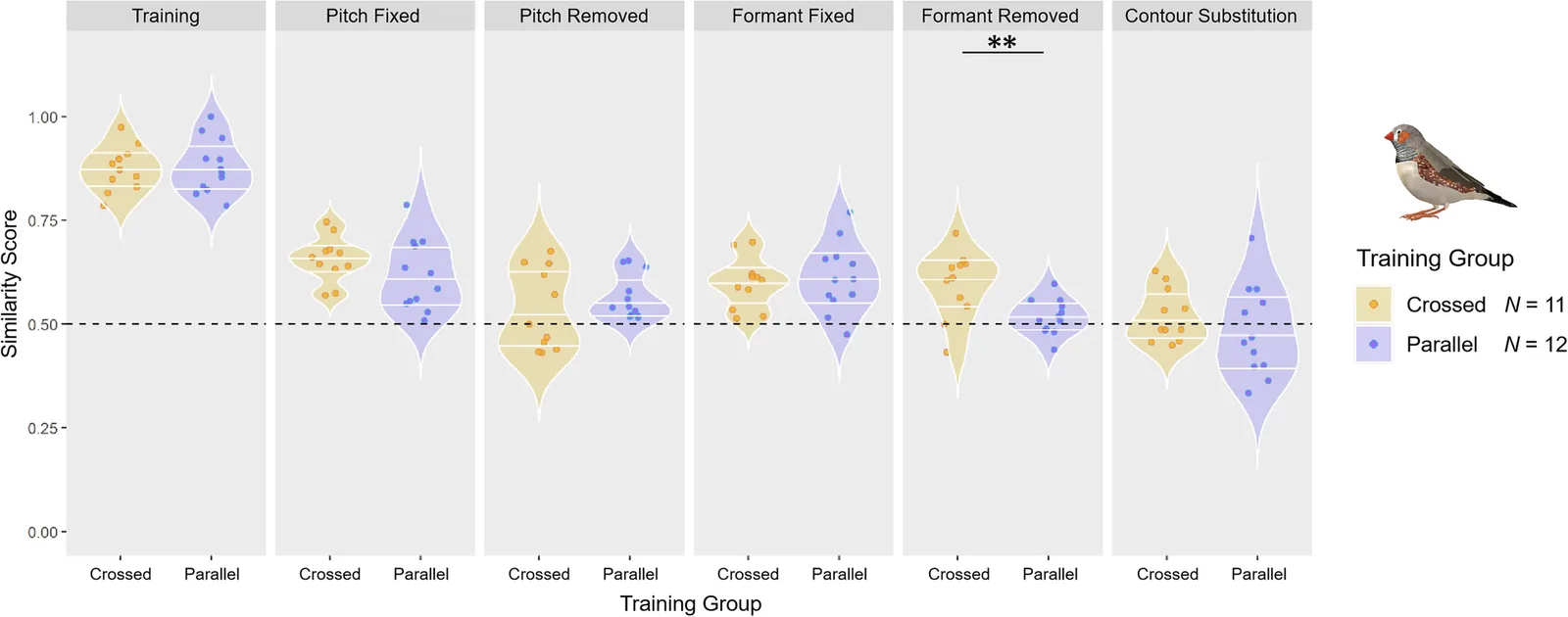
Similarity scores for training and modified stimuli across the two training groups in zebra finch experiment. Significant differences between the training groups (Crossed-Contours, *N* = 11 biologically independent animals; Parallel-Contours, *N* = 12 biologically independent animals) for the responses to each test stimulus are indicated: ** refers to a significant difference of 0.001 < *P* ≤ 0.01, and for non-indicated comparisons *P* value is > 0.05; exact *P* values for all statistical comparisons underlying the figure are provided in Table S2. The violin plots are kernel density estimations. Median, 1st and 3rd quartiles are indicated by the white horizontal lines. Statistical comparisons were performed using two-sided contrasts of estimated marginal means from GLMMs. No adjustment for multiple comparisons was applied because comparisons between the two training groups were prespecified planned contrasts for each test stimulus. The Crossed-Contours group showed significantly higher similarity scores than the Parallel-Contours group for formant-removed stimuli (GLMM estimated marginal means contrast, *P* = 0.0024). For the Contour-Substitution probes, a similarity score approaching 1 indicates classification according to the formant contour (and consequently opposite to the pitch contour) of the training stimulus, whereas a similarity score approaching 0 indicates classification according to the pitch contour of the training stimulus. The Horizontal dashed line represents chance level, which was 50% for both tasks.

Similarity scores did not differ significantly between the Parallel- and Crossed-Contours groups for most probe stimuli, except for the *formant-removed* version (Fig. 3), where the Crossed-Contours group showed higher similarity scores (difference = 0.347 ± 0.114, *P* = 0.002; Table S2).

When either pitch or formant contour was fixed (*pitch-fixed* or *formant-fixed* versions; Fig. 2), zebra finches still classified the probe stimulus as similar to its corresponding training stimulus, indicating that either feature independently supported recognition when the other was held constant (Fig. 3). Rather than favoring one dimension, zebra finches appear to integrate both pitch contour and formant contour during sequence discrimination. The birds’ low similarity scores on the *contour-substitution* version, in which a modified stimulus could be treated as either training stimulus A or B depending on whether pitch or formant was attended to, shows that zebra finches respond randomly when the two features provide contradictory information, confirming that both cues are treated as comparably important during recognition.

Post hoc pairwise comparisons (FDR-adjusted) revealed that both groups exhibited significantly higher similarity scores for the non-rewarded training stimuli compared to any of the five modified versions (all *P* < 0.0001). This further supports that both pitch and formant contributed to discrimination, as every modification led to a marked reduction in similarity scores regardless of training group (Table S2).

Both training groups showed significantly higher similarity scores for the *pitch-fixed* stimuli than for the *formant-removed* (Crossed-Contours group: *P* < 0.05; Parallel-Contours group: *P* = 0.0001), *pitch-removed* (Crossed-Contours group: *P* < 0.0001; Parallel-Contours group: *P* < 0.05), and *contour-substitution* (both groups: *P* < 0.0001) stimuli. Likewise, similarity scores for the *formant-fixed* stimuli were higher than for the *contour-substitution* stimuli (Crossed-Contours group: *P* < 0.01; Parallel-Contours group: *P* < 0.0001). Despite the comparable relevance of pitch and formant in both experimental groups, subtle group-specific differences in relative cue weighting indicated some context-dependent modulation around a generally balanced integration of both cues. In the Crossed-Contours group, *pitch-fixed* stimuli elicited higher similarity scores than *formant-fixed* stimuli (*P* < 0.05), and *formant-removed* stimuli elicited higher similarity scores than *pitch-removed* (*P* < 0.05) or *contour-substitution* stimuli (*P* < 0.01). In the Parallel-Contours group, similarity scores were significantly higher for *formant-fixed* than for *formant-removed* stimuli (*P* < 0.001), and higher for *pitch-removed* than for *contour-substitution* stimuli (*P* < 0.01). In this group, there were also trends toward higher similarity scores for *pitch-removed* than *formant-removed*, as well as for *formant-fixed* than *pitch-removed* stimuli (both *P* = 0.08).

#### Response rates

Response rates showed no significant differences between training groups for any stimuli (Fig. S1a) and the response rate patterns broadly mirrored similarity scores. Both groups exhibited significantly higher response rates for the non-rewarded training stimuli compared to any of the five modified versions (all *P* < 0.0001; Table S2) and responded more frequently to *formant-fixed* and *pitch-fixed* than to the *formant-removed* stimuli (Crossed-Contours group: both *P* < 0.01; Parallel-Contours group: both *P* < 0.001) and less often to *pitch-removed* than to any other modified stimuli (Crossed-Contours group: both *P* < 0.0001; Parallel-Contours group: both *P* < 0.01). Within-group contrasts further showed that birds in the Crossed-Contours group responded more to *pitch-fixed* and *formant-fixed* than to *contour-substitution* (both *P* < 0.05), whereas birds in the Parallel-Contours group responded more to *contour-substitution* than to *formant-removed* stimuli (*P* < 0.001). Notably, both groups showed relatively high response rates to the *contour-substitution* stimuli despite their low similarity scores.

#### LogRatios

LogRatio analyses (Fig. S2a, Table S5) also largely converged between the two training groups. In the Crossed-Contours group, responses to *pitch-removed* and *contour-substitution* did not significantly deviate from chance (logRatio ≈ 0), while all other stimuli were reliably discriminated above chance. In the Parallel-Contours group, chance-level performance was observed for *formant-removed* and *contour-substitution* stimuli, with significant discrimination above chance for all other stimuli.

### Robust pitch bias in humans

As in the zebra finches, we evaluated performance in the human online task using similarity scores and response rates, complemented by logRatio values. Across both training groups, pitch consistently emerged as the dominant cue. However, we observed substantial variation in the response to probe stimuli lacking pitch information, which was driven by musical experience rather than by the specific alignment of pitch and formant contours during training. Given this variability and the uneven distribution of musical experience between training groups (see Methods), we therefore focus on analyses that explicitly account for musical experience.

#### Similarity scores

**Fig. 4:**
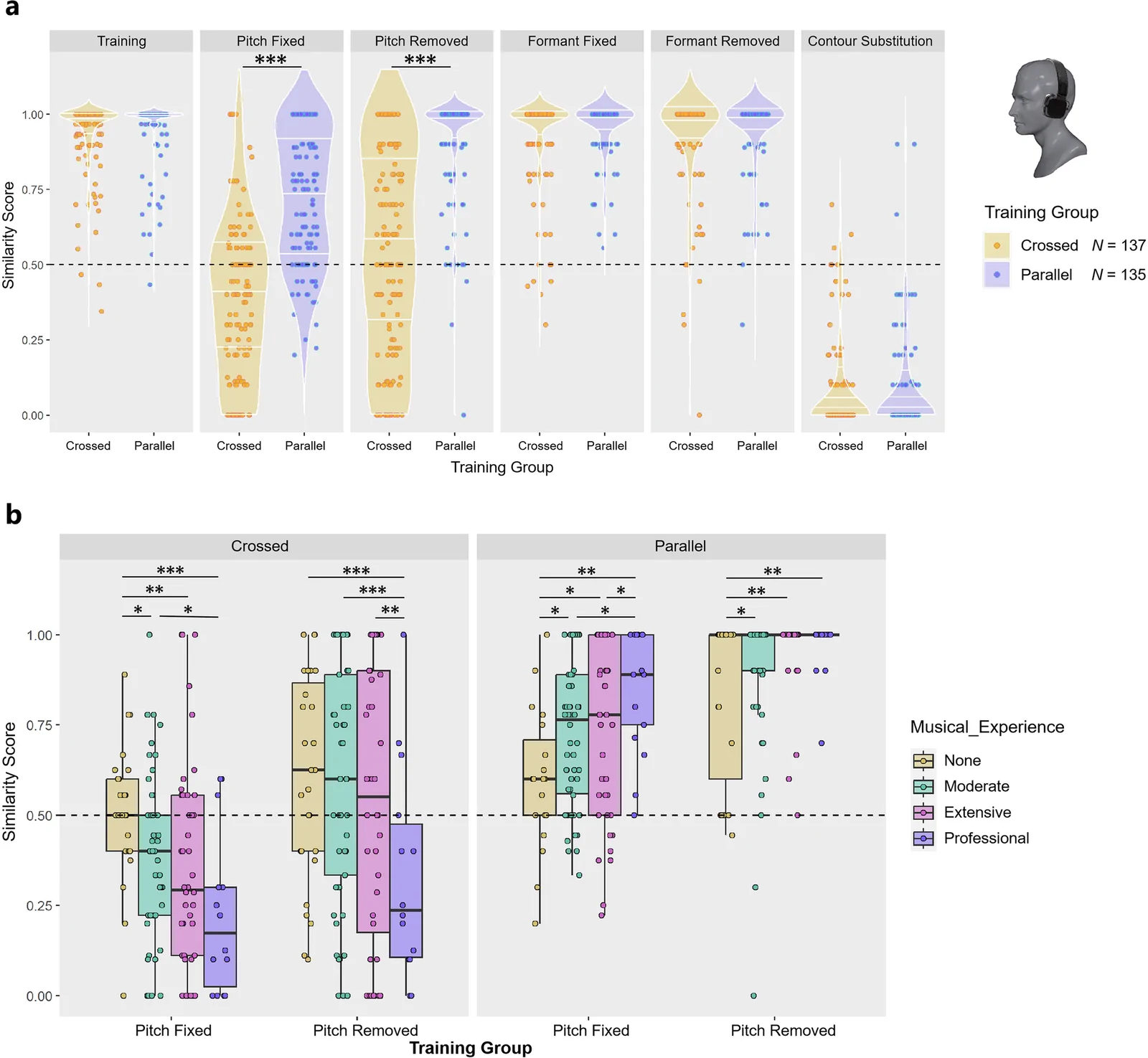
Similarity scores in human online task. **a** Comparison of similarity scores between two training groups (Crossed-Contours, *N* = 137 biologically independent human participants; Parallel-Contours, *N* = 135 biologically independent human participants). Statistical tests were two-sided. Statistical comparisons were performed using two-sided contrasts of estimated marginal means from generalized linear mixed-effects models (GLMMs). No adjustment for multiple comparisons was applied because these comparisons were planned contrasts; exact *P* values for all statistical comparisons underlying panel a are provided in Table S3. The Crossed-Contours group showed significantly higher similarity scores than the Parallel-Contours group for pitch-fixed and pitch-removed stimuli (GLMM estimated marginal means contrasts, both *P* < 0.0001). For the Contour-Substitution probes, a similarity score approaching 1 indicates classification according to the formant contour (and consequently opposite to the pitch contour), whereas a similarity score approaching 0 indicates classification according to the pitch contour. **b** Comparison of similarity scores among participants with different levels of Musical Experience when responding to pitch-fixed and pitch-removed stimuli. Participants were categorized according to musical experience as None (Crossed-Contours, *N* = 27 biologically independent human participants; Parallel-Contours, *N* = 19 biologically independent human participants), Moderate (Crossed-Contours, *N* = 51 biologically independent human participants; Parallel-Contours, *N* = 56 biologically independent human participants), Extensive (Crossed-Contours, *N* = 45 biologically independent human participants; Parallel-Contours, *N* = 43 biologically independent human participants), or Professional (Crossed-Contours, *N* = 14 biologically independent human participants; Parallel-Contours, *N* = 17 biologically independent human participants). Statistical comparisons were performed using two-sided post-hoc pairwise comparisons of estimated marginal means following generalized linear mixed-effects models (GLMMs), with false discovery rate (FDR) correction for multiple comparisons; exact adjusted *P* values for all 24 pairwise comparisons underlying panel b are provided in Table S4. Significant differences in both panels are indicated as follow: *** refers to a significant difference of *P* ≤ 0.001, ** refers to a significant difference of 0.001 < *P* ≤ 0.01, and * refers to a significant difference of 0.01 < *P* ≤ 0.05, for non-indicated comparisons *P* value is > 0.05. The violin plots in panel a represent kernel density estimations, with white horizontal lines indicating the median, first, and third quartiles. The box plots in panel b display the median (central horizontal line) and first and third quartiles (lower and upper box boundaries), with whiskers extending to the most extreme observations within 1.5 × the interquartile range. Individual points represent individual participants. The box plots therefore show the distribution of individual participant similarity scores and do not represent standard errors, standard deviations, or confidence intervals. The horizontal dashed lines in both panels indicate the chance level, set at 50% for both tasks.

Irrespective of training group and musical experience, the highest similarity scores were shown towards the *formant-fixed* and *formant-removed* stimuli (Fig. 4a; Table S3). Similarity scores for these formant-based modifications did not differ significantly from those for the *non-rewarded training* stimuli (Table S3). These results indicate a strong dominance of pitch in the human classification of the test stimuli.

When results were collapsed across musical experience, similarity scores for the *pitch-fixed* and *pitch-removed* stimuli (both *P* < 0.0001) differed between the Parallel- and Crossed-Contours groups (Fig. 4a). These group-level differences were accompanied by a large spread in responses, indicating substantial heterogeneity across participants, and appear to be strongly modulated by musical experience (Figs. 4b; S2c). In the Crossed-Contours group, participants with no musical experience (“None”) showed the highest similarity scores for these pitch-based modifications. With increasing musical experience, similarity scores decreased (even falling below 0.5) indicating that greater musical experience was associated with stronger biases toward classifying probe stimulus A as similar to training stimulus B (Fig. 4b; Table S4). These participants, but not the “None” experienced ones, were influenced by the formant pattern of the probe stimuli. They seem sensitive to the direction of changes in the formant pattern in the probe stimuli and treat this as if it provided information about pitch changes. In the Parallel-Contours group, the pattern for stimuli lacking pitch information was reversed. Participants with “None” musical experience showed lower similarity scores than all other experience levels (all *P* < 0.05), which were close to 0.5 (or slightly above), again confirming the dominant role of pitch cues. In contrast to the Crossed-Contours group, increasing musical experience in the Parallel-Contours group resulted in progressively higher similarity scores for the stimuli lacking reliable pitch information (all *P* < 0.05; Fig. 4b; Table S4). Thus, here too, musically experienced (but not “None” experienced) participants appeared to respond to the direction of formant changes in the probe stimuli when pitch cues were unavailable.

#### Response rates

Both training groups were similar in the number of responses to all probe stimuli, apart from their response to the *pitch-removed* stimuli. Across training groups, there were no significant differences among the four levels of musical experience in response rates to any of the probe stimuli (Fig. S1c; Table S4), indicating that response rates were not modulated by musical experience or training condition. When data were collapsed across musical experience, participants in the Crossed-Contours group responded significantly less often to the *pitch-removed* stimuli than those in the Parallel-Contours group (*P* < 0.001; Fig. S1b; Table S3). This pattern suggests that participants in the Crossed-Contours group were more likely to treat the *pitch-removed* stimuli as unclassifiable. Additionally, response rates to the *contour-substitution* stimuli did not differ from those for the training stimuli in either group (Table S3). This pattern suggests that listeners tended to treat the *contour-substitution* stimuli as equivalent to one of the training stimuli, regardless of training condition.

#### LogRatio

LogRatio analyses closely mirrored the similarity-score results, providing converging evidence for a robust pitch bias in humans and an experience-dependent use of secondary cues. Across training groups and musical experience levels, participants reliably discriminated formant-based modifications (*formant-fixed* and *formant-removed* stimuli) at above-chance levels (logRatio > 0), whereas the *contour-substitution* stimuli elicited a significant bias toward “mismatching response” (logRatio < 0) (Fig. S2b; Table S6), despite comparable response rates. This pattern again confirms the dominant role of pitch and indicates that the *contour-substitution* stimuli were treated as perceptually equivalent to the opposite training stimuli.

Cue-use patterns diverged most clearly for the pitch-based modifications. For the *pitch-fixed* stimuli, participants in the Parallel-Contours group showed above-chance discrimination across all levels of musical experience. In contrast, in the Crossed-Contours group, participants with “None” musical experience performed at chance (logRatio ≈ 0), whereas those with higher levels of experiences showed a systematic bias toward “mismatching response”. A similar stratification emerged for the *pitch-removed* stimuli. The Parallel-Contours group again discriminated reliably regardless of experience, while in the Crossed-Contours group, participants with “None” or “Moderate” experience performed above chance, those with “Extensive” experience showed chance-level performance, and those with highest experience (“Professional”) exhibited a strong bias toward “mismatching response” (Fig. S2c; Table S6).

Notably, formant contours were perceivable by both highly and minimally experienced listeners: even participants with the least musical experience showed above-chance discrimination when pitch information was absent (*pitch-removed*), despite failing to discriminate stimuli with ambiguous pitch cues (*pitch-fixed*). The asymmetry between the Parallel-Contours and the Crossed-Contours group indicates that it may be easier to recruit formant information as a secondary cue in evaluating the probe stimuli if pitch and formant changed in the same direction across the training stimuli, with its significance again being affected by musical experience (Fig. S2c). Overall, the logRatio analysis reinforces the conclusion that human listeners prioritize pitch, while musical experience promotes more flexible cue weighting when pitch cues are unreliable.

## Discussion

Our results identify both shared and species-specific strategies for extracting sequential structure from complex tones. Across zebra finches and humans, the training regime (Parallel versus Crossed contours) exerted little influence on overall discrimination, indicating that the directional alignment of pitch and formant trajectories during learning did not, by itself, organize how listeners deployed cues. Instead, performance in both species was best explained by how each auditory system weights and recruits co-occurring dimensions when they are available, degraded, or in conflict.

Zebra finches showed robust multi-cues integration. Their performance was consistent with an additive integration strategy in which either pitch or formant information could support discrimination when the other dimension was held constant. This pattern suggests that finches treated both trajectories as informative components of a single sequential pattern, rather than as mutually exclusive alternatives. In other words, they did not appear to prioritize one contour as the dominant “reference” for sequence identity. Our study is the first to systematically test the trade-off between pitch and formants for discriminating harmonic complex tone sequences. The finding that zebra finches attend to both pitch and formant patterns accords with earlier studies demonstrating that altering the pitch or the formant structure in speech-like or artificial stimuli and songs disrupted the recognition of familiar stimuli^26,34,36,44,45^. Nevertheless, the failure of the zebra finches to classify stimuli in which the spectral envelope of the formants is still present, but pitch information is disrupted, is not due to a general insensitivity to spectral envelope cues. Rather, zebra finches are able to exploit envelope information when pitch-related structure is degraded, as demonstrated by responses to vocoded vowel-like stimuli^26^ and the preserved behavioral efficacy to male calls despite missing *f*₀ and most harmonics^7^. Together, these findings suggest context-dependent cue weighting in zebra finches, with envelope cues possibly more relevant for short, call-like signals, whereas discrimination of sequential sounds or songs relies more on attending to other spectro-temporal details (e.g., see Dooling & Prior^23^; also see Geberzahn & Derégnaucourt^46^). In this respect, our data differ from those obtained with starlings, in which Bregman et al.^22^ observed recognition of noise-vocoded tone sequences. Whether this indicates a genuine difference in species-specific perceptual strategies, with zebra finches more sensitive to pitch disruption of artificial tone sequences than starlings, or might be due to differences in methodology or stimulus details, remains to be explored.

In contrast to zebra finches, humans exhibited a strong and pervasive pitch dominance. Their performance was largely governed by pitch whenever it was available—an effect that was apparent regardless of musical background. Musical experience mattered most when pitch information became unreliable. Although formants are especially important in speech perception, the present stimuli were not speech sounds and did not contain the phonetic context in which formant information normally supports vowel identification. It is therefore more likely that listeners processed the stimuli as sequences of changing acoustic events rather than through specifically speech-related mechanisms. From this perspective, changes in either *f*₀ or formant structure may have provided a common cue to the direction of spectral movement across the sequence^13^. When pitch information was unavailable, formant contours could therefore serve as an alternative representation of the learned relational pattern, supporting classification based on shared acoustic contours rather than on specific musical or linguistic structures^5^. Importantly, musically trained listeners did not simply fall back on a memorized rule about the direction of the trained formant contour; if they had, one would expect stronger formant-consistent responding in the Crossed-contours condition. Instead, the observed pattern points in the opposite direction and points to a different mechanism: experienced listeners demonstrated a functional substitution of perceptual dimensions to preserve the “relational” structure of the learned pitch contour by extracting frequency-change relations from an alternative spectral dimension (the formant contour) and mapping those relations onto pitch-based templates acquired during training. This account also explains the asymmetry across training conditions. In the Parallel-contours group, formant changes in the *pitch-fixed* and *pitch-removed* probes were aligned with the trained pitch direction, providing a useful surrogate cue and yielding higher similarity scores among experienced listeners; in the Crossed-contours group, the same surrogate conflicts with the trained pitch direction, resulting in opposite similarity scores in musically trained listeners. Minimally trained listeners, despite being strongly pitch-oriented, still performed modestly above chance when pitch was removed, consistent with a baseline sensitivity to spectral contour change that was typically subordinated when pitch cues were present.

These findings refine what musical expertise contributes to human multidimensional sequence perception. Experience supported the use of secondary dimensions to preserve learned relational structure when the primary cue was degraded—without eliminating cross-dimensional interference or reversing the basic asymmetry in cue learning when pitch co-occurs with other dimensions. This framing aligns with evidence that musicians are not uniformly less susceptible than nonmusicians to interference from task-irrelevant dimensions such as spectral centroid or pitch^39^, and with reports that even trained listeners can remain vulnerable to certain timbral interference during pitch judgments^43,47^, despite occasional demonstrations of enhanced resilience (e.g., see Pitt^48^). More broadly, musical training often strengthens pitch processing yet does not reliably improve sensitivity to other spectral dimensions that are not explicitly cultivated during training^39^, and it has not shown consistent links to cross-dimensional sequence-processing efficiency (e.g., see Cousineau et al.^49^).

Formal musical training was associated with greater flexibility in the use of secondary cues. However, the strong preference for pitch across all experience groups shows that musical expertise alone cannot account for the human pattern. Apart from a potential species-specific biological bias, the bias may also be shaped more broadly by prolonged exposure to pitch-structured sounds, including singing, instrumental music, and other culturally common auditory experiences. The preference for pitch, as observed here, hence most likely reflects an interaction between species-specific auditory predispositions and accumulated experience. Taken together, these findings suggest that auditory experience, including but not limited to formal musical training, promotes the flexible recruitment of secondary cues when the dominant cue is unreliable. At the same time, it does not eliminate the underlying asymmetry that favors pitch when multiple dimensions are available. This bias in dimensional mapping may be uniquely human (or at least strongly species-specific): whereas human listeners can transfer pitch-contour relations to other acoustic dimensions^13^, zebra finches showed no evidence of comparable cross-dimensional remapping.

Rather than reflecting either biology or experience alone, the present findings suggest that cue-weighting strategies emerge through interactions between species-specific auditory predispositions and accumulated experience. Zebra finches underwent a longer acclimation and pre-training period than humans, but these phases were intended primarily to establish the operant procedure rather than the discrimination itself; differential reinforcement of the training stimuli began only during the formal discrimination phase. Human participants, by contrast, brought a lifetime of exposure to pitch-rich auditory environments, which may have reinforced the weighting of pitch as a dominant cue. The species differences observed here are therefore best interpreted as arising from the combined influence of auditory predispositions and experience. Future studies that systematically manipulate pre-exposure across species will help disentangle the respective contributions of task familiarity and species-specific auditory processing.

These cross-species differences sharpen mechanistic accounts of sequential sound perception by demonstrating that even species capable of perceiving pitch and formant can differ in how these dimensions are weighted and recruited under the present experimental conditions, consistent with distinct cue-weighting architectures—one that integrates multiple spectral dimensions, and another that defaults to pitch and recruits alternative cues primarily under constraint. More broadly, these species differences place boundary conditions on accounts of sequential sound perception by showing that extracting contour-based structure can be achieved through distinct computational architectures shaped jointly by species-specific auditory organization and accumulated auditory experience. Comparative studies based on production or preference (e.g., see Logan et al.^50^) may therefore reveal different patterns from those examining how species perceive and represent acoustic patterns.

## Methods

### Experimental model and study participant details

#### Zebra finches

A total of thirty-one zebra finches (*Taeniopygia guttata*) (13 males and 18 females) were introduced into the experiment, and were randomly assigned to one of the two experimental groups. Of these, twenty-three zebra finches (12 males and 11 females) completed the full experiment, whereas eight birds did not reach criterion performance during discrimination training and were excluded before probe testing (5 in the Parallel-Contours group and 3 in the Crossed-Contours group). The birds that completed the experiment were trained and tested at the age of 334 ± 47 days post-hatching (dph), (age males: M = 366, SD = 37, age females: M = 299, SD = 26). All birds originated from the in-house breeding colony at Leiden University. Birds lived in single-sex aviaries and transferred to standard laboratory cages (two same-sex birds per cage) for one-week acclimation prior to the experiment; full housing conditions are identical to those reported in our previous studies^44,45^. In the end, twenty-three experimental birds successfully completed the pre-training (see below) and were included for the final experiment: the Parallel-Contours group consisted of twelve birds, the Crossed-Contours group consisted of eleven birds (6 males and 6 females in Parallel-Contours group, 6 males and 5 females in Crossed-Contours group; age Parallel-Contours group: M = 316, SD = 33, age Crossed-Contours group: M = 353, SD = 51). None had prior task or stimulus experience.

The experiment and procedures adhered to the European and Dutch legislation on animal experimentation and were approved by the Animal Experiment Committee (DEC; AVD number 1060020197507) All procedures were performed according to the guidelines of the Leiden University Committee for Animal Experimentation (see also Ning et al.^45^ for details of the ethical note).

#### Human participants

A total of 272 adult human participants (120 males, 141 females, 5 identifying as other gender, and 6 preferring not to disclose their gender) participated in an online listening categorization task analogous to the operant discrimination paradigm used in the zebra finch experiment. Participants were randomly assigned to the Parallel-Contours group (*N* = 135) or the Crossed-Contours group (*N* = 137) (see also Table 1 for the sex distribution in each group). Participants were recruited from students at the University of Amsterdam, Leiden University, and social media platforms. Participation was on an entirely voluntary basis, and no compensation was provided. All participants were adults (ages 19–80 years; median age = 34 years) and reported normal hearing. The study consisted of a minimal-risk, fully online listening categorization task. The study protocol was reviewed and approved by the Ethics Committee of the Faculty of Humanities of the University of Amsterdam. No separate approval/reference number was issued for this online behavioral study. The study was conducted in accordance with the ethical standards of the Declaration of Helsinki. All participants provided informed consent prior to participation.

**Table 1:** Overview of the training groups

Training group	Sequence series	Zebra finches (*N*)	Humans (*N*)	Sequence of tonal units
Crossed_Contours	Series 1	3♂ + 3 ♀	31♂ + 36♀ + 1U	430 Hz(2.6 kHz)-360Hz(3.1 kHz)-290Hz(3.6 kHz)-220Hz(4.1 kHz)-150Hz(4.6 kHz)
Crossed_Contours	Series 2	3♂ + 2 ♀	35♂ + 28♀ + 3U + 3X	405 Hz(2.1 kHz)-330Hz(2.7 kHz)-255Hz(3.3 kHz)-180Hz(3.9 kHz)-105Hz(4.5 kHz)
Parallel_Contours	Series 1	3♂ + 3 ♀	22♂ + 44♀ + 1X	150 Hz(2.6 kHz)-220Hz(3.1 kHz)-290Hz(3.6 kHz)-360Hz(4.1 kHz)-430Hz(4.6 kHz)
Parallel_Contours	Series 2	3♂ + 3 ♀	32♂ + 33♀ + 2U + 1X	105 Hz(2.1 kHz)-180Hz(2.7 kHz)-255Hz(3.3 kHz)-330Hz(3.9 kHz)-405Hz(4.5 kHz)

In each training group, the tone sequences were arranged in the same way (the pitch and formant contours of a stimulus were either going in the parallel or crossed direction) but differed in *f*₀ and Formant values of their complex tones. The numerical values between the “-” indicates the *f*₀ and Formant values of each complex tone (e.g., “430 Hz(2.6 kHz)” stands for *f*₀ = 430 Hz and Formant peak = 2.6 kHz). The values of tonal units for tone sequence are shown in this table, with training stimulus A presented as an exemplar. While training stimulus B is not displayed in this table, note that the values of tonal units for training stimulus B were identical to those of training stimulus A, with the only difference being that its tonal units were arranged in the reverse order of training stimulus A. See text for details.

### Reporting summary

Further information on research design is available in the Nature Portfolio Reporting Summary linked to this article.

## Method details

### Zebra finch experiment

#### Operant conditioning cage

Zebra finches were trained and tested individually in an operant conditioning cage (Fig. 1a), described previously^44,45^.

#### Procedure

A Go-Left/Go-Right paradigm was used for both training and testing in the zebra finch experiment. The procedure comprised five phases: acclimation, pre-training, discrimination training, transition, and probe testing. A brief overview of the procedure is provided here (see also Fig. 1e), as full procedural details of the songbird Go-Left/Go-Right experiment have been reported previously^44,45^. During the acclimation phase, birds were introduced to the experimental cage and allowed to freely explore the apparatus while becoming familiar with the playback of the training stimuli and the location of the food hatch. During the subsequent pre-training phase, birds familiarized the sensor–sound–reward contingencies required for the Go-Left/Go-Right task. At this stage, birds were not yet required to discriminate between the two training stimuli or associate them with different response sensors; differential reinforcement of Stimulus A versus Stimulus B began only during the discrimination-training phase. In the discrimination training phase, birds learned to peck the central sensor (S1) to initiate sound playback, and then to choose the appropriate left or right sensor (S2 or S3) depending on the sound. Correct choices were reinforced with a 15-seconds food hatch opening; incorrect responses led to a 1-second light-off signal. Once birds reached criterion performance (discrimination score > 0.75 for three consecutive days), they entered the transition phase, where 20% of training trials were no longer reinforced. Birds that maintained discrimination during this phase proceeded to the probe testing phase, in which 20% of trials involved unreinforced presentations of probe stimuli (modified versions of the trained sounds) randomly interspersed among the reinforced training trials. Each probe stimulus was presented 40 times per subject before testing concluded.

#### Stimuli

##### Training stimuli

The training stimuli in the songbird experiment (as well as in human online task) were 4 stimulus pairs (2 sequence series/pairs for each training group, as shown in Table 1), each pair consisted of two harmonic sequences (each made up of five complex tones) that differ in both pitch and formant direction. In both experiments, two training groups were defined based on the stimuli used during training. For the Crossed-Contours group, the training stimuli consisted of pairs of complex tones in which the pitch and formant contours of a single stimulus varied in opposite directions, for example, the pitch contour of training stimulus A decreased across the tone sequence, while its formant contour increased (Fig. 2a). For the Parallel-Contours group, the training stimuli consisted of pairs of complex tones in which both the pitch and formant contours of a single stimulus varied in the same direction—either both increasing or both decreasing (Fig. 2a).

In each stimulus pair, one stimulus (e.g., training stimulus A) featured a formant-ascending pattern, for example, “2.6kHz- 3.1kHz- 3.6kHz- 4.1kHz- 4.6 kHz”. This formant-ascending pattern increased the amplitude (+30 dB) within specific frequency bands of each complex tone. These enhanced bands fell within the hearing threshold of zebra finches (see Okanoya & Dooling^51^) and humans, typically peaking around 3.5–4.0 kHz. In contrast, the complex tones of the other stimulus (e.g., training stimulus B) followed a formant-descending pattern, for instance, “4.6kHz- 4.1kHz- 3.6kHz- 3.1kHz- 2.6 kHz”. Similarly, one sequence featured a pitch-ascending pattern, such as “150Hz- 220Hz- 290Hz- 360Hz- 430 Hz”, while the other exhibited a pitch-descending pattern, for instance, “430Hz- 360Hz- 290Hz- 220Hz- 150 Hz”. Across sequence series within each training group, stimuli were arranged to share the same contour structure but differed in *f*₀ and formant values to prevent the selected value set from accidentally coinciding with frequencies that might hold specific biological relevance for the birds. When played, sequences within each stimulus pair were RMS-normalized (calculated over the total duration of a stimulus) to equate overall intensity and prevent response bias driven by amplitude differences.

The training stimuli were synthesized, normalized and filtered using Praat (version 6.1.12) and Audacity (version 2.3.0). To synthesize a sound unit, the first step was to construct a complex tone with a defined *f*₀ value (e.g., 150 Hz as the *f*₀) by choosing the function “Create Sound as tone complex” (to create a sound combining the *f*₀ and its constituent harmonics occurring at integer multiples of the *f*₀) in Praat. Secondly, the complex tone was manipulated by the Effect “Fade in/Fade out” and its peak amplitude was Normalized to “-50 dB” by Audacity. Additionally, to create a formant for the unit, the amplitude located in a particular frequency range (e.g., the Formant peak located in 2.6 kHz) was amplified (Width 1 kHz, Gain 15 dB x2) by applying the “Parametric EQ” function (an effect plugin) in Audacity. Afterwards these complex tones were combined into a single sequence using Praat, with each tone (duration: 0.21 s) separated by a silence of 0.05 s, resulting in a sequence with a duration of 1.25 s. All stimuli were low-pass filtered (below 8 kHz).

##### Probe stimuli

To assess the relative contributions of pitch and formant contours, birds (as well as human participants) were tested with probe versions of each training sequence (Fig. 2b). Across both the Crossed-Contours and the Parallel-Contours training groups, probe stimuli were generated using identical manipulations that selectively altered pitch or formant information. Praat and Audacity were used to edit each original training stimulus to produce these probe versions:

(i) *pitch-fixed*—In this manipulation, all pitches were made equal to the *f*₀ of the middle unit, while preserving the formant contour of the original training stimulus. For example, a sequence with five harmonic units featuring “*f*_*0*_ = 290 Hz” and arranged in a formant-ascending pattern of “2.6kHz- 3.1kHz- 3.6kHz- 4.1kHz- 4.6 kHz”. This adjustment was achieved by modifying the “Frequency (Hz)” parameter within the “Parametric EQ” function in Audacity.

(ii) *pitch-removed*—This modification maintains the spectral envelope (both its shape and position) of the training stimulus, but averages the energy within specific frequency bands, thus removing any harmonic structure. For this we used the Matt Winn’s Praat vocoded script (http://www.mattwinn.com/praat/vocode_all_selected_v45.txt) to synthesize a *pitch-removed* version of training stimuli. The script was set to divide cut-off frequency bandwidths equally for 30 bands contiguous with smooth transitions (From lowCornerFreq 100 Hz to highCornerFreq 8000 Hz).

(iii) *formant-fixed*—In this stimulus a single, fixed formant was added to the training stimulus, using the formant value of the middle unit of the sequences. As such it preserved the pitch contour, while all elements have the same spectral envelope. This manipulation was accomplished by assigning a same “Frequency (Hz)” value (the “Parametric EQ” function in Audacity) to harmonic units of different *f*_*0*_.

(iv) *formant-removed*—The purpose of this manipulation was to create stimuli where all frequency bands have the same energy, while keeping the *f*_*0*_ values identical to training stimuli. The construction of the *formant-removed* version followed the same procedure as that of the training stimuli, except for omitting the Formant synthesis step. This ensured that the *formant-removed* version maintained the same characteristics (tone sequences with increasing/decreasing pitch contour) as the training stimuli, yet without any formant.

(v) *contour-substitution*—In this modification, the formant contour of the training stimulus was altered by adjusting the formant frequencies of each harmonic unit and arranging them in a reversed order compared to the original training stimulus. This modification aimed to combine the formant contour of one training stimulus with the pitch contour of the other training stimulus from the same training pair. For example, a modified version of training stimulus A would share the formant contour of training stimulus A while sharing the pitch contour of training stimulus B. In the analysis, the responses to this “ambiguous” contour-substitution stimulus are compared to the training stimulus that shares the same formant contour.

Both training and probe stimuli have not been heard before by the subjects.

### Online listening categorization task

#### Procedure

The human online listening task was engineered as a “digital Skinner box” (Fig. 1c) to maintain strict procedural symmetry with the songbird experiment (Fig. 1a), and was carried out through the following link: https://www.mcg.uva.nl/humansvszebrafinches/. We utilized an experimental procedure where participants automatically learned the underlying stimulus-response contingencies through visual ‘positive’- and ‘negative’- feedback (Fig. 1d), directly mirroring the food-access and light-off reinforcement used with the zebra finches (Fig. 1b). Participants were asked to situate themselves in a quiet environment to avoid distractions and began with adjusting the volume of a calibration sound to a comfortable level, ensure stimuli were audible and confirm that they had normal hearing (see *Consent*). In many cases, apart from those requiring precise control over absolute sound levels, online experiments have been shown to produce results indistinguishable from those obtained under more controlled laboratory conditions, provided that precautions are taken to ensure sound presentation quality and participant compliance^52^.

The human online listening categorization task consisted of three phases: participants first completed an introduction phase, providing consent and background information. They then entered the training phase, where they learned to discriminate between two sounds by clicking one of two colored buttons after hearing a playback, receiving smiley, sad, or confused face feedback according to their responses. After reaching a performance criterion, participants entered the probe testing phase, where they categorized modified stimuli mixed among the training stimuli.

##### Introduction phase (Pre-experiment)

Before the experiment is really starting, the computer screen will be showing an instruction “*This is a listening experiment in which you have to respond to short sound sequences*”, followed by a page with a request for consent. After that, the participant can click on “*Agree and continue*” to accept the agreement. This will be followed by the Instructions “*We need to know a bit about you*”. Here they have to type in their age, and select one of the options form the Gender Categories: ‘Male (M)’, ‘Female (F)’, ‘Other (X)’, ‘Prefer not to disclose (U)’. This phase is closed with the request “And finally, please select your level of musical expertise.” and they will be asked to choose one of the following options: ‘None’, ‘Moderate’, ‘Extensive’ or ‘Professional’.

##### Training phase

At the start of the experiment, the computer screen displayed a full-screen with a playback (speaker) icon in the center, accompanied by the instruction: “*The experiment will now begin. Click to start a sound sequence*”. When participants clicked the playback icon, a sound sequence was presented. After each sound sequence finished playing, two response buttons become clickable, one blue and one brown, positioned side by side below the playback icon.

Participants responded to sound sequence in each trial by clicking one of two colored buttons (blue or brown). No explicit instructions were given; instead, participants learned through feedback that selecting the correct button (e.g., blue for stimulus A, brown for stimulus B) elicited a smiley face, whereas choosing the opposite mapping (e.g., brownbrown for stimulus A, blue for stimulus B) elicited a sad face. During the training phase, the two stimuli A and B were presented in random order with equal probability. The assignment of button color to stimulus A or B was randomized across participants but fixed within an individual to control for potential effects of color–position associations.

The response window was limited to 3 s after playback; if no response was given within this period, a confused face appeared on screen, and the trial was recorded as a *no response*. After each trial, the playback button is refreshed, and clicking it initiated the next trial. Training phase consisted of 20 short sound sequences (stimulus A and stimulus B each played for 10 times in randomized order). Participants advanced to the testing phase once they reached criterion performance (≥ 8 correct responses out of 10 trials). Otherwise, an additional block of 10 trials was presented until the criterion was achieved. Upon reaching the criterion, participants were shown an instruction message: “*You are entering the main phase of the experiment. From now on you will only occasionally get feedback on your responses. Simply try to keep responding to the sound sequences as you did before*”. In practice, nearly all participants (270 of 272) reached the training criterion during the initial 20-trial training block. Only two participants (both from the “Extensive” musical_experience category) required one additional 10-trial training block before proceeding to the probe testing phase.

##### Probe testing phase

The testing phase comprised five novel versions of the training stimuli intermixed with the original training stimuli. The response window was shortened to 2.5 s. Each of the training stimuli (A and B) was presented 15 times, with 10 trials providing feedback (smiley/sad face) and 5 trials providing no feedback. Each stimulus from the five novel versions (each consisting of a pair, stimulus A and B; 10 stimuli in total) was presented five times without feedback. This resulted in a total of 80 trials in this phase. Stimuli were presented in randomized order, with trials of training stimuli interspersed among trials of novel stimuli to help maintain participants’ memory of the training contingencies. The task lasted approximately 20 min, but varied depending on the number of training blocks completed to reach the training criterion.

#### Stimuli

Human participants completed a listening categorization task analogous to the zebra finch experiment. Stimuli were identical to the bird experiment (training and probe stimuli). All stimulus materials are publicly available on the Open Science Framework (OSF) (DOI: 10.17605/OSF.IO/ZXDBJ).

## Quantification and statistical analysis

### Zebra finch experiment

In the zebra finch experiment (as well as in the human online listening task), reactions to the various probe stimuli were categorized into three types: a “matching response” (i.e., identifying the modified version of training stimulus A as A, and the modified version of training stimulus B as B), an “mismatching response” (i.e., choosing B when the stimulus was a modification of sound A, and vice versa), and a “no response” (i.e., failing to respond by selecting an answer). Two primary measures were derived: the Similarity Score, calculated as the proportion of “matching response” out of all “matching response + mismatching response” (Similarity Score = Trial_Matching / (Trial_Matching + Trial_Mismatching)); and the Response rate, calculated as the proportion of trials in which participants responded (i.e., made either a correct or incorrect response) out of all 40 presentations of each probe stimulus (Response_Rate = (Trial_Matching + Trial_Mismatching) / (Trial_Matching + Trial_Mismatching + Trial_NoResponse)).

We used Generalized Linear Mixed-effects Models (GLMMs) to examine the discrimination of various test sounds by the subjects. All model analyses were conducted in Rstudio (R package *lme4*)^53^. We calculated the Similarity Score and the Response Rate based on the counts of “matching response”, “mismatching response”, and “no response”, combining the response counts to (variants of) Training stimuli A and B, (using the function *cbind*, R package *mice*)^54^, and used these two indicators as response variables in GLMMs in R (using the function *glmer*, R package *lme4*)^53^.

In the zebra finch experiment, we used “Training_Group” (Parallel-Contours or Crossed-Contours training pairs), “Test_Stimuli”, “Sequence_Series” and the interaction between “Training_Group” and “Test_Stimuli” as covariates in the full model with “Bird_ID” as the random factor and a binomial error structure of the “Similarity score” and the “Response rate”. The best model was selected based on the corrected Akaike Information Criterion (AICc), using the *dredge* function from the R package *MuMIn*^55^. By default, the model with the lowest AICc value was considered the best; however, if “Training_Group”, “Test_Stimuli”, or their interaction were not included in the best model, we retained them in the final model regardless, as they were variables of primary interest (Table S1). To assess the effects and significance of covariates, we ran the final models and, where applicable, conducted post hoc pairwise comparisons using estimated marginal means contrast (*emmeans* function, *lsmeans* package)^56^, applying false discovery rate (FDR) correction for multiple comparisons^57^. For the above models, the counts of the reactions to modifications of both sound A and sound B were combined.

To determine whether individual probe stimuli were discriminated above chance (50%), we additionally assessed the ratio of “matching response” to “mismatching response” (Trial_Matching / Trial_Mismatching). Specifically, we analyzed the log-transformed ratio, log(Trial_Matching / Trial_Mismatching), hereafter referred to as the Log Ratio, using a binomial generalized linear model (GLM) to test whether this value differed significantly from 0. A ratio of 1 (i.e., log(1) = 0) corresponds to equal probabilities of “matching response” and “mismatching response” (i.e., 50% chance performance). Thus, comparing the outcomes of the Binomial GLM to 0 is comparing the results to the 50% chance for a response that identifies a probe stimulus A as training stimulus A (and probe stimulus B as training stimulus B as well).

### Online listening categorization task

#### Data collection

In this experiment, participants categorize novel stimuli according to the acoustic feature they used in the training. To control for any bias arising from color preference, for part of the participants, responses on the blue button were scored as correct for stimulus A and responses on the brown button as correct for stimulus B; for the remaining participants, this mapping was reversed.

All data were documented as an Excel / .csv file, including: (i) Stimulus code – label of the presented stimulus; (ii) Response – button selected; (iii) Reaction time – interval between stimulus onset and button click; (iv) Musical experience – None (Crossed-Contours: *N* = 27; Parallel-Contours: *N* = 19), Moderate (Crossed-Contours: *N* = 51; Parallel-Contours: *N* = 56), Extensive (Crossed-Contours: *N* = 45; Parallel-Contours: *N* = 43), or Professional (Crossed-Contours: *N* = 14; Parallel-Contours: *N* = 17); (v) Age and gender – age entered numerically and further categorized into four age stages (Age_Stages): Early Adulthood (18–29 years), Early Middle Age (30–44 years), Late Middle Age (45–59 years), and Seniorhood (60 years and older); and (vi) Gender, coded as Male (*M*), Female (*F*), Other (*X*), or Undisclosed (*U*).

#### Statistical analysis

As in the zebra finch experiment, reactions to the various probe stimuli in the human online task were categorized into three types: (i) *matching response* (identifying a modified version of training stimulus A as A, or training stimulus B as B); (ii) *mismatching response* (choosing B when the probe stimulus was a modification of training stimulus A, and vice versa); and (iii) *no response* (failing to respond within the allotted time).

For the statistical analyses, we derived two main measures. The Similarity Score was defined as the proportion of *matching response* out of all reactions (Similarity_Score = Trial_Matching/(Trial_Matching + Trial_Mismatching)). The Response Rate was defined as the proportion of trials in which participants responded (i.e., made a choice either *matching response* or *mismatching response*) out of all presentations of each probe stimulus (Response_Rate = (Trial_Matching + Trial_Mismatching)/(Trial_Matching + Trial_Mismatching + Trial_NoResponse)).

Because some participants gave no responses or always responded to certain stimulus versions, leading to zeros in the denominator of the Similarity Score or the Log Ratio, we applied a regularization adjustment to avoid this bias in the binomial models. Specifically, given that training stimuli was played three times more than novel probe stimuli (30 times comparing to 10 times), we added 3 to both the *matching response* and *mismatching response* counts (or to both *response* and *NoResponse*) for training stimuli, and 1 to both count categories for all other probe stimuli. This modified Agresti–Coull adjustment, implemented in R using the mutate() function from the *dplyr* package^58^, ensured stable estimates across all three model types (Similarity Score, Response Rate, and LogRatio).

In the GLMM analyses, following the same approach as in the zebra finch experiment, Training_Group, Test_Stimuli, and the Training_Group × Test_Stimuli interaction were included as fixed effects. In the full model, we included Musical_Experience, Age_Stages, Gender, and the three-way interaction Training_Group × Test_Stimuli × Musical_Experience as additional covariates. Participant_ID was included as a random factor. Age_Stages and Gender were excluded from the final models, as model selection based on AICc (*MuMIn* package)^55^ indicated that these factors did not improve model fit (Table S1). Model significance was assessed by running the final models, and post hoc pairwise comparisons were conducted on estimated marginal means (emmeans function, *lsmeans* package)^56^, applying false discovery rate (FDR) correction for multiple comparisons^57^. To assess whether individual probe stimuli were discriminated above chance (50%), we also calculated the Log Ratio, and tested whether this value differed significantly from 0 using binomial generalized linear models.

### Statistics and reproducibility

The biological replicate in both experiments was defined as an individual subject (one zebra finch or one human participant). Repeated trials collected from the same individual were treated as repeated measurements and were not considered independent replicates. The zebra finch experiment included 23 birds (Parallel-Contours: *n* = 12; Crossed-Contours: *n* = 11), and the human experiment included 272 adult human participants (Parallel-Contours: *n* = 135; Crossed-Contours: *n* = 137). All stimulus materials, behavioural procedures, and analysis criteria were predefined and applied consistently within each species. The same acoustic stimuli and behavioural paradigm were used across species, with only the response interfaces adapted to the requirements of the zebra finch and human experiments.

Statistical analyses were conducted separately for the two species because of differences in behavioural implementation. Probe responses were analyzed using Similarity Scores, Response Rate, and Log Ratio as response-based measures. Mixed-effects models were used where appropriate to account for repeated measurements within individuals, and post hoc comparisons were corrected for multiple testing where applicable. Statistical significance was assessed at α = 0.05. Detailed statistical procedures, model structures, and post hoc comparisons are described in the corresponding Methods sections.

## Supplementary information

Transparent Peer Review file

Supplementary Information

Description of Additional Supplementary Files

Supplementary Data 1

Supplementary Data 2

Reporting Summary

## Data Availability

Supplementary Figs. S1 and S2 and Supplementary Table S1–S6 are available in the Supplementary Information. The source data for Fig. 3 are provided in Supplementary Data 1 and include raw behavioural scores from the 23 zebra finches (also related to Figs. S1a and S2a). The source data for Fig. 4 are provided in Supplementary Data 2 and include raw behavioural scores from the 272 human participants (also related to Figs. S1b,c and S2b,c). All stimulus materials generated in this study are publicly available via the Open Science Framework (OSF): https://doi.org/10.17605/OSF.IO/ZXDBJ^59^

## Code availability

No original code was generated in this study. Any additional information required to reanalyze the data reported in this paper is available from the lead contact, Zhi-Yuan Ning (zhiyuanning@hotmail.com), upon request.
