# Supplementary material for: Divergent processing strategies for auditory sequence recognition in humans and zebra finches (Taeniopygia guttata)
Source: Commun Biol. 2026 Oct 13;9:1281. doi: 10.1038/s42003-026-11035-9 (PMC13661893; doi:10.1038/s42003-026-11035-9)
Supplement: Supplementary file 1 [file 42003_2026_11035_MOESM1_ESM.pdf]

nature portfolio

# Peer Review File

# Divergent Processing Strategies for Auditory Sequence Recognition in Humans and Zebra Finches (Taeniopygia guttata)

Corresponding Author: Dr Zhi-Yuan Ning

**This file contains all reviewer reports in order by version, followed by all author rebuttals in order by version.**

Version 0:

Reviewer comments:

Reviewer #1

(Remarks to the Author)
In this excellent manuscript, the authors investigate how zebra finches and humans categorize acoustic stimuli based on pitch and formant patterns, with particular emphasis on what each species does when the stimuli are ambiguous. The introduction sets up the experiment well and outlines key concepts for a broad audience, and the results accord with previous research while extending some key details and providing a direct species comparison using nearly identical tasks. I have just a few comments that I think will improve the presentation:

Clarity of sample sizes could be improved in the Results and/or Figure legends (e.g., xx responses to xx probes by xx subjects).

A bit more explanation of the training paradigm would be useful in the text. This is in the figures, but never explicitly described in the text. Specifically, on l. 118 it indicates that subjects heard tone sequences, but does not mention that they were trained to associate different sequences with a different button before the test stimuli.

It is indicated that there are some exclusion criteria based on performance for the zebra finches, but no indication of how many birds were excluded due to this. Would limiting the human participants to those that achieved at least as well on training as the zebra finches lead to any differences in conclusions? I don't see any depiction of the accuracy on training stimuli based on music skill.
Discussion should highlight that the pool of human participants was relatively narrow (including a remarkably high level of professional and extensively trained musicians), cautioning over-interpretation across the species. Indeed, I assume these musicians were largely trained in Western contexts, and this should be acknowledged as a narrowing of what "musical" training may lead to (it is possible that different musical trainings can lead to different types of sensory biases).

Suggestions to improve figure clarity:
Fig. 2: Key could indicate what red and blue lines indicate
Fig. 3,4: Indicating the species in the figure. Also indicate that "similarity scores" of 1 for contour substation reflect similarity based on formant.

Reviewer #2

(Remarks to the Author)
This paper describes a carefully-designed set of experiments that compare auditory processing of sound sequences between humans and a songbird, the zebra finch (ZF). The authors' objective was to "to assess how zebra finches and humans weigh co-occurring (whether congruent or incongruent) patterns of acoustic dimensions when discriminating sequences of complex tones." To this end, they created a similar operant paradigm for both human and ZF subjects, and asked them to discriminate the exact same stimuli. ZFs were tested in behavioral chambers in the laboratory and humans who visited an online website were tested in a virtual replica.
Training "tones" were polyharmonic sounds presented as a sequence of 5 sounds that either ascended or descended in

fundamental frequency or formant timbre. These 2 parameters could either progress in the same (congruent) or opposite (incongruent) directions across the sequence. After subjects reached criterion in the training phase, they then heard various kinds of probe stimuli that shared one or more features with the training sounds to assess which features were being used. “The responses to each probe stimulus were assigned a similarity score reflecting how closely the probe stimulus was perceived to resemble either of the two training stimuli”

A semantic point (Lines 141-2, 111, 167): we actually don’t know how the stimuli were “perceived” by either humans or ZFs. We only know whether and how the probe stimuli were classified in the behavioral paradigm. Please use neutral wording.

The experimental design is sound, the writing is clear and the figures are appropriate. The results provide a reasonably clear picture of performance similarities and differences between human and ZF subjects. While ZFs are able to equally weight formant or frequency transitions in classifying probe stimuli with respect to training exemplars, human subjects appear to rely primarily on pitch transitions to classify stimuli, although there was considerable individual variability. This led to a further sub-grouping of human subjects according to musical experience as a possible factor; however, pitch remained the dominant cue across sub-groups. A further observation was that humans had a tendency to classify formant slopes across sounds as if they were pitch slopes, and this was more likely among the musically-experienced, leading to poor classification performance when formants were not the relevant parameter.
While the empirical findings are robust and convincing, I am unsure about whether they demonstrate a fundamental species difference. So I think the discussion should be softened on this point for 2 reasons.
First, despite the attempt to make the task as similar as possible for both species, if I understand the procedures, there were significant differences in initial exposure to the stimuli. The full paradigm was not described; the reader was referred to methods of earlier papers: Ning et al (2023 and 2024). The birds had an “acclimation” phase during which they were repeatedly exposed to the A and B sounds on which they would be subsequently trained. Then, during a “pre-training” period, pecks to these stimuli were reinforced, although it’s not clear exactly which stimuli were played during pre-training. While those first phases may be necessary to teach the bird how to use the apparatus, birds received what appears to be a large number of exposures to the stimuli, although not specifically reinforced for A vs. B until formal training. So the stimuli were first experienced as environmental sounds (perhaps startling but not salient) that subsequently got differentially reinforced.
Humans were not told what the task was, but, as soon as they started responding, they got differential reinforcement and training. So birds got a lot more neutral exposure than humans. I wonder what would happen if the humans had had as much unreinforced exposure to the training sounds as the birds had, e.g. as background sounds during some foreground task.
Second, what did humans think the task was? The site asked them to “beat the birds”. The clear shift in central frequency (whether F0 or formant timbre) and the contrast between A and B suggested that the task was about the change across stimuli, either ascending or descending. To understand better, I went to the test site myself and tried training. To my untrained ear, the stimuli either seemed to go up or down regardless of which parameter was changing. I am not musically-experienced, but I made a lot of training errors because I heard the formant changes as pitch changes (even though I knew the experimental design). Formants are an important cue for speech, but this was not a speech task and these “vowels” were not embedded between consonants, so speech mechanisms were unlikely to have been engaged. Humans may have heard this as a melody.

Overall, the results say something real about the birds’ unbiased use of cues, but I think the human data are confused not necessarily by explicit musical training but by a lifetime of exposure to singing in school (Do-Re-Mi etc..) and musical instruments. So the human tendency to focus on pitch may say something about culture, not necessarily about a deep species difference. The authors do acknowledge this possibility when they write “extracting contour-based structure can be achieved through distinct computational architectures shaped by biology and experience.” However, the discussion needs to say more about the role of experience: both the effects of pre-exposure in the birds and the effects of culture in the humans.

Version 1:

Reviewer comments:

Reviewer #1

(Remarks to the Author)
I appreciate the authors' careful responses to the reviews, and am happy to recommend publication.

Reviewer #2

(Remarks to the Author)
The authors have clarified the methods, improved data presentation, and tempered the discussion of species differences. The revised manuscript is much improved and represents a useful contribution.

**Open Access** This Peer Review File is licensed under a Creative Commons Attribution 4.0 International License, which permits use, sharing, adaptation, distribution and reproduction in any medium or format, as long as you give appropriate credit to the original author(s) and the source, provide a link to the Creative Commons license, and indicate if changes were made.
In cases where reviewers are anonymous, credit should be given to 'Anonymous Referee' and the source.
The images or other third party material in this Peer Review File are included in the article's Creative Commons license, unless indicated otherwise in a credit line to the material. If material is not included in the article's Creative Commons license and your intended use is not permitted by statutory regulation or exceeds the permitted use, you will need to obtain permission directly from the copyright holder.
To view a copy of this license, visit https://creativecommons.org/licenses/by/4.0/

# Point-by-point response to reviewers

**Date:** 03$^{rd}$ August 2026
**Manuscript Number:** COMMSBIO-26-3969-T
**Title of Article:** Following the Tune: Divergent Processing Strategies for Auditory Sequence Recognition in Humans and a Songbird
**Name of Corresponding Authors:** Zhi-Yuan Ning; & Henkjan Honing
**Email Address of Corresponding Authors:** zhiyuanning@hotmail.com; H.J.Honing@uva.nl

Dear Editor (Luke Remage-Healey),

Thank you for the opportunity to revise our manuscript in *Communications Biology*. We sincerely appreciate you and both reviewers for careful evaluation of our manuscript and for constructive and thoughtful comments, and are delighted that both reviewers found the study of interest and regarded the experimental findings robust. We have carefully considered every comment and have revised the manuscript accordingly. All changes to the manuscript have been highlighted or indicated using tracked changes. The revisions include clarifications of the experimental procedures, additional reporting of sample sizes and exclusion criteria, improvements to the figures, and a more balanced discussion of the potential contributions of biology and experience to the observed species differences. Below, we provide a detailed point-by-point response to each comment.

On behalf of the authors

Yours sincerely,
Zhi-Yuan Ning

## Response to Reviewer #1

**General comments:**

**In this excellent manuscript, the authors investigate how zebra finches and humans categorize acoustic stimuli based on pitch and formant patterns, with particular emphasis on what each species does when the stimuli are ambiguous. The introduction sets up the experiment well and outlines key concepts for a broad audience, and the results accord with previous research while extending some key details and providing a direct species comparison using nearly identical tasks. I have just a few comments that I think will improve the presentation.**

**Authors:** We sincerely thank the reviewer for the thoughtful and constructive evaluation of our manuscript. We have carefully considered all of the reviewer's suggestions and believe that the revisions have substantially improved the clarity and interpretation of the manuscript. Our point-by-point responses are provided below.

**Detailed comments:**

**1. Clarity of sample sizes could be improved in the Results and/or Figure legends (e.g., xx responses to xx probes by xx subjects).**

**Authors:** We thank the reviewer for this helpful suggestion. To improve the clarity of the sample sizes, we have revised the figure legends presenting the behavioral results to report the sample sizes of subjects included in each plot explicitly (see legends of Fig. 3, Fig. 4, Fig. S1, and Fig. S2). For analyses further subdivided by musical experience, the sample size of each musical-experience category within each training group is now also provided in the corresponding figure legend (see legends Fig. 4, Fig. S1, and Fig. S2). We believe this adaptation makes the sample sizes readily accessible while keeping the Results section concise.

**2. A bit more explanation of the training paradigm would be useful in the text. This is in the figures, but never explicitly described in the text. Specifically, on l. 118 it indicates that subjects heard tone sequences, but does not mention that they were trained to associate different sequences with a different button before the test stimuli.**

**Authors:** We thank the reviewer for this helpful suggestion. We agree that the original text did not sufficiently explain the training paradigm before introducing the probe-test results. We have therefore revised the manuscript to briefly describe that both zebra finches and human participants first learned to associate the two training sound sequences (Stimulus A and Stimulus B) with distinct responses through trial-by-trial feedback before proceeding to the probe testing phase (see lines 90-95). This addition provides readers with a clearer overview of the experimental design without duplicating the more detailed procedural description presented in the Methods.

**3. It is indicated that there are some exclusion criteria based on performance for the zebra finches, but no indication of how many birds were excluded due to this. Would limiting the**

**human participants to those that achieved at least as well on training as the zebra finches lead to any differences in conclusions? I don't see any depiction of the accuracy on training stimuli based on music skill.**

**Authors:** We thank the reviewer for these thoughtful suggestions. We have addressed each point separately below.

(1) *Number of excluded zebra finches*

We have now clarified the number of birds that did not reach the training criterion and were therefore excluded from subsequent testing. In total, 31 zebra finches (13 males and 18 females) entered the experiment, of which 23 (12 males and 11 females) successfully completed the full procedure. Specifically, 12 of 17 birds in the Parallel-Contours group and 11 of 14 birds in the Crossed-Contours group reached the training criterion and proceeded to probe testing, while the remaining eight birds (five in the Parallel-Contours group and three in the Crossed-Contours group) did not reach criterion performance and were therefore excluded prior to testing. This information has now been added to the Methods (see lines 345-350, and lines 354-356).

(2) *Human training performance*

Regarding the reviewer's question about human training performance, nearly all participants reached the training criterion during the initial training block. Specifically, only two participants (both belonging to the "Extensive" musical-experience category; refer to participants with ID 1673 & 1675 in the file "Supplementary Data 3") required one additional block of ten training trials before meeting the training criterion. Given this, we do not expect their inclusion to influence the conclusions of the study. We have clarified this point in the Methods (see lines 525-528).

(3) *Training accuracy & musical experience*

We thank the reviewer for the suggestion. We have clarified the Methods to indicate that nearly all participants (270 of 272) reached the training criterion during the initial 20-trial training block. Because training performance was effectively at ceiling and exhibited negligible variation across musical-experience groups, we did not consider a separate analysis of training accuracy to be informative. Instead, our analyses focus on the probe phase, which was specifically designed to assess cue weighting after successful acquisition of the discrimination task.

**4. Suggestions to improve figure clarity:**

**Fig. 2: Key could indicate what red and blue lines indicate**

**Authors:** We thank the reviewer for this helpful suggestion, and have added a tiny legend in panel (a) of Fig. 2.

**Fig. 3,4: Indicating the species in the figure. Also indicate that "similarity scores" of 1 for contour substation reflect similarity based on formant.**

**Authors:** We thank the reviewer for this helpful suggestion. We have revised Figures 3 & 4 to indicate explicitly whether the data are from zebra finches or humans by adding a small icon of a

zebra finch or a human to the upper-right corner of corresponding figures. In addition, we have clarified in the legends for the Contour-Substitution probes in figures 3 & 4 (see lines 957-959, and lines 972-974). We believe these revisions improve the readability of the figures and make the interpretation of the Contour-Substitution results more intuitive.

## Response to Reviewer #2

### General comments:

**This paper describes a carefully-designed set of experiments that compare auditory processing of sound sequences between humans and a songbird, the zebra finch (ZF). The authors' objective was to "to assess how zebra finches and humans weigh co-occurring (whether congruent or incongruent) patterns of acoustic dimensions when discriminating sequences of complex tones." To this end, they created a similar operant paradigm for both human and ZF subjects, and asked them to discriminate the exact same stimuli. ZFs were tested in behavioral chambers in the laboratory and humans who visited an online website were tested in a virtual replica.**

**Training "tones" were polyharmonic sounds presented as a sequence of 5 sounds that either ascended or descended in fundamental frequency or formant timbre. These 2 parameters could either progress in the same (congruent) or opposite (incongruent) directions across the sequence. After subjects reached criterion in the training phase, they then heard various kinds of probe stimuli that shared one or more features with the training sounds to assess which features were being used. "The responses to each probe stimulus were assigned a similarity score reflecting how closely the probe stimulus was perceived to resemble either of the two training stimuli"**

**The experimental design is sound, the writing is clear and the figures are appropriate. The results provide a reasonably clear picture of performance similarities and differences between human and ZF subjects. While ZFs are able to equally weight formant or frequency transitions in classifying probe stimuli with respect to training exemplars, human subjects appear to rely primarily on pitch transitions to classify stimuli, although there was considerable individual variability. This led to a further sub-grouping of human subjects according to musical experience as a possible factor; however, pitch remained the dominant cue across sub-groups. A further observation was that humans had a tendency to classify formant slopes across sounds as if they were pitch slopes, and this was more likely among the musically-experienced, leading to poor classification performance when formants were not the relevant parameter.**

**Authors:** We sincerely thank the reviewer for the careful and constructive evaluation of our manuscript. We are pleased that the reviewer found the experimental design to be sound, the writing clear, and the figures appropriate, and that the empirical findings were described as 'robust and convincing' while providing a clear picture of the similarities and differences in cue weighting between humans and zebra finches. We particularly appreciate the reviewer's thoughtful discussion of the possible mechanisms underlying the observed human pattern, including the role of cross-dimensional cue transfer and prior auditory experience. These comments have led us to clarify several aspects of the manuscript, including the description of the behavioural paradigm, the interpretation of the human data, and the respective contributions of biology and accumulated experience to the observed cross-species differences. We believe these adaptations have substantially strengthened the manuscript.

**A semantic point (Lines 141-2, 111, 167): we actually don't know how the stimuli were "perceived" by either humans or ZFs. We only know whether and how the probe stimuli were classified in the behavioral paradigm. Please use neutral wording.**

**Authors:** We thank the reviewer for this crucial semantic point. We agree that our behavioural paradigm measures stimulus classification rather than subjective perception. Accordingly, we have revised the manuscript throughout to replace wording "perceived" with more neutral terms where appropriate (see lines 107-108, line 125, and lines 931-932).

**While the empirical findings are robust and convincing, I am unsure about whether they demonstrate a fundamental species difference. So I think the discussion should be softened on this point for 2 reasons.**
**First, despite the attempt to make the task as similar as possible for both species, if I understand the procedures, there were significant differences in initial exposure to the stimuli. The full paradigm was not described; the reader was referred to methods of earlier papers: Ning et al (2023 and 2024). The birds had an "acclimation" phase during which they were repeatedly exposed to the A and B sounds on which they would be subsequently trained. Then, during a "pre-training" period, pecks to these stimuli were reinforced, although it's not clear exactly which stimuli were played during pre-training. While those first phases may be necessary to teach the bird how to use the apparatus, birds received what appears to be a large number of exposures to the stimuli, although not specifically reinforced for A vs. B until formal training. So the stimuli were first experienced as environmental sounds (perhaps startling but not salient) that subsequently got differentially reinforced.**

**Authors:** We thank the reviewer for this thoughtful comment. We agree that zebra finches necessarily experienced a longer acclimation and pre-training phases than human participants because they first had to learn the operant procedure before formal discrimination training could begin. To make this clearer, we have adapted and expanded the description of the acclimation and pre-training phases in the Methods and now explicitly state that differential reinforcement of the two training stimuli began only during the formal discrimination-training phase (see lines 389-393).

**Humans were not told what the task was, but, as soon as they started responding, they got differential reinforcement and training. So birds got a lot more neutral exposure than humans. I wonder what would happen if the humans had had as much unreinforced exposure to the training sounds as the birds had, e.g. as background sounds during some foreground task.**

**Authors:** Regarding the possibility that the different amount of pre-exposure contributed to the observed species differences, we notice that virtually all human participants (270 of 272; 99.3%) met the discrimination criterion during the initial 20-trial training block, with only two participants requiring one additional block of 10 trials before entering the probe phase (both belonging to the "Extensive" musical-experience category; refer to participants with ID 1673 & 1675 in the file "Supplementary Data 3"). This indicates that participants rapidly acquired the stimulus–response associations. We therefore consider it unlikely that the principal findings reflect differences in learning the discrimination itself. Instead, the probe phase was specifically designed to assess which acoustic cues participants relied upon after successful acquisition of the task. Nevertheless, we agree that differences in pre-exposure history constitute a procedural

difference between species and have acknowledged this limitation and soften relate claim in the Discussion of the cross-species comparison (see lines 319–329, and line 332).

**Second, what did humans think the task was? The site asked them to "beat the birds". The clear shift in central frequency (whether F0 or formant timbre) and the contrast between A and B suggested that the task was about the change across stimuli, either ascending or descending. To understand better, I went to the test site myself and tried training. To my untrained ear, the stimuli either seemed to go up or down regardless of which parameter was changing. I am not musically-experienced, but I made a lot of training errors because I heard the formant changes as pitch changes (even though I knew the experimental design). Formants are an important cue for speech, but this was not a speech task and these "vowels" were not embedded between consonants, so speech mechanisms were unlikely to have been engaged. Humans may have heard this as a melody.**

**Authors:** We thank the reviewer for this thoughtful observation and appreciate that they took the time to experience the online task. Interestingly, the reviewer reports the same tendency to interpret formant contours as pitch contours that was observed behaviourally in many of our participants. We agree that the stimuli were more likely to have been interpreted as sequential melodic patterns than as speech-like sounds. Accordingly, we have expanded the Discussion to note that, although the stimuli contained formant manipulations, they were not speech sounds and were therefore unlikely to have primarily engaged speech-specific processing mechanisms. We now discuss the possibility that listeners spontaneously interpreted the five-tone sequences as melodic contours, allowing formant contours to function as surrogate cues for the relational pitch structure acquired during training (see lines 272-279).

**Overall, the results say something real about the birds' unbiased use of cues, but I think the human data are confused not necessarily by explicit musical training but by a lifetime of exposure to singing in school (Do-Re-Mi etc..) and musical instruments. So the human tendency to focus on pitch may say something about culture, not necessarily about a deep species difference.**

**Authors:** We agree that explicit musical training alone is unlikely to fully explain the human pattern observed in the present study. Accordingly, we have expanded the Discussion to distinguish formal musical training from broader accumulated auditory experience. Specifically, we now discuss how lifelong exposure to pitch-based auditory environments may contribute to the development of the observed pitch bias. At the same time, because robust pitch dominance was observed across all musical-experience groups, we then conclude that the human pattern is likely to reflect an interaction between accumulated auditory experience and species-specific auditory predispositions, rather than either factor alone (see lines 306–316).

**The authors do acknowledge this possibility when they write "extracting contour-based structure can be achieved through distinct computational architectures shaped by biology and**

**experience." However, the discussion needs to say more about the role of experience: both the effects of pre-exposure in the birds and the effects of culture in the humans.**

**Authors:** We thank the reviewer for this valuable suggestion and agree that the role of experience should be discussed more explicitly. We have therefore revised the Discussion to acknowledge that zebra finches underwent a longer acclimation and pre-training period than humans, while clarifying that these phases were designed primarily to establish the operant procedure rather than the discrimination itself; differential reinforcement of the two training stimuli began only during the formal discrimination-training phase. We also discuss how humans enter the experiment with extensive prior exposure to pitch-based auditory environments. Accordingly, we now emphasize that the observed cross-species differences are most consistent with an interaction between species-specific auditory predispositions and accumulated experience, rather than reflecting biology or experience in isolation. We also note that future studies systematically manipulating pre-exposure across species would help to disentangle these respective contributions (see lines 319–329, and line 337-339).

## Additional minor revisions

**Authors:** In addition to the revisions made in response to the reviewers' comments, we made several further refinements during the revision process. We also implemented numerous adjustments throughout the manuscript in response to the editorial comments, including revisions to the manuscript title, reporting standards, figure and table presentation, data

availability statements, supplementary material organization, and other formatting requirements to ensure compliance with the journal's guidelines. We revised terminology throughout the manuscript to improve clarity, precision, and consistency, including the description of probe-stimulus classification, similarity scores, and experimental procedures.

We also identified a minor inconsistency in the schematic of the experimental procedures (Fig. 1). In the original figure, the outcome following a missed response was labeled "*No feedback*" for both zebra finches and humans. However, in the human task, participants who failed to respond within the response window were actually presented with a confused-face icon before the next trial began. To remove this inconsistency and to use terminology that is applicable to both species, we have replaced this label with "*Timeout*" in both Fig. 1b and Fig. 1d, and revised the legend to use identical terminology throughout (see line 918, and line 928). During revision, we also improved the Figure 1 legend to better describe the experimental procedures and ensure consistency with the Methods. Specifically, we clarified the *feedback* associated with correct and incorrect responses (see line 918), and corrected the response window for the human online task from "*15 seconds*" to "*3 seconds*" (see line 927).

Finally, following discussion among the authors, we added a brief clarification in the Discussion regarding recent comparative work on acoustic preferences and production (Logan et al. 2026). Specifically, we now note that comparisons based on production or preference may reveal different patterns from those examining perceptual mechanisms (see lines 337–339).

We believe these additional revisions improve the clarity, consistency, and interpretation of the manuscript.
