# Supplementary material for: Divergent processing strategies for auditory sequence recognition in humans and zebra finches (Taeniopygia guttata)
Source: Commun Biol. 2026 Oct 13;9:1281. doi: 10.1038/s42003-026-11035-9 (PMC13661893; doi:10.1038/s42003-026-11035-9)
Supplement: Supplementary file 2 [file 42003_2026_11035_MOESM2_ESM.pdf]

Fig. S1: Response rate of trials.

a

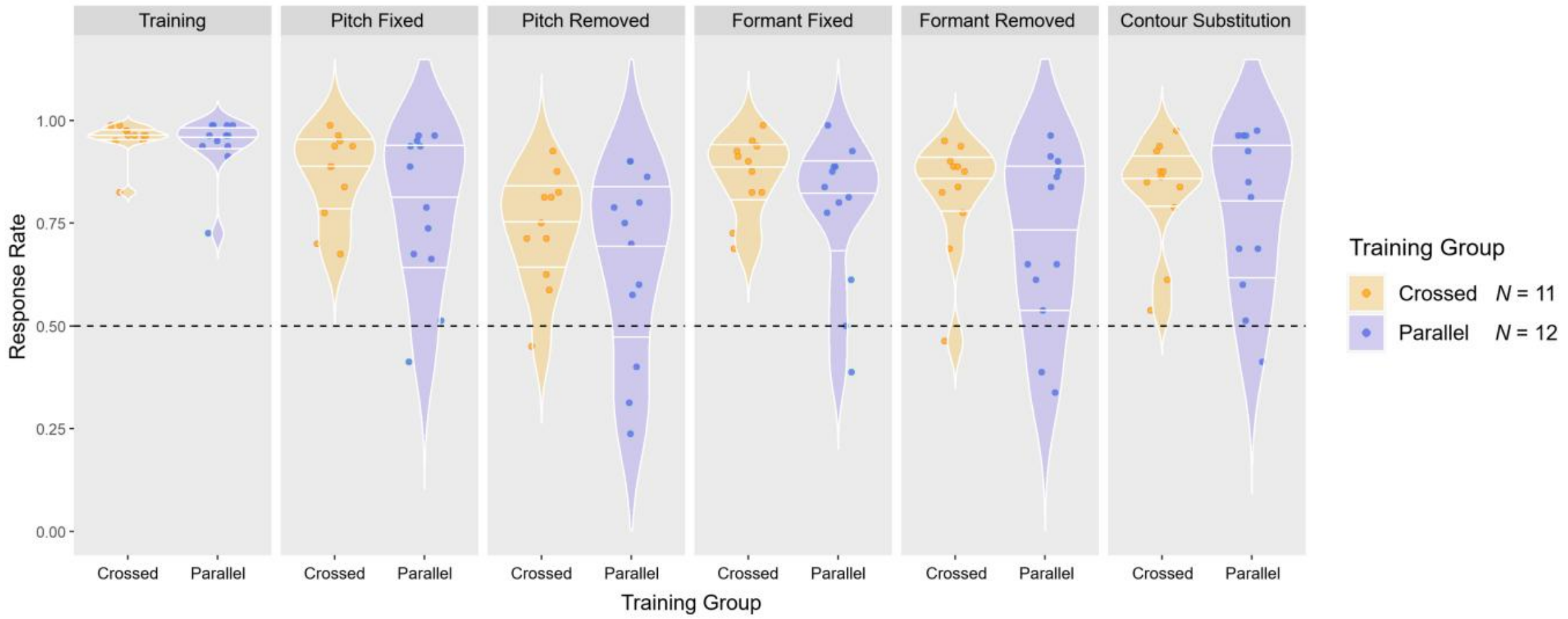

b

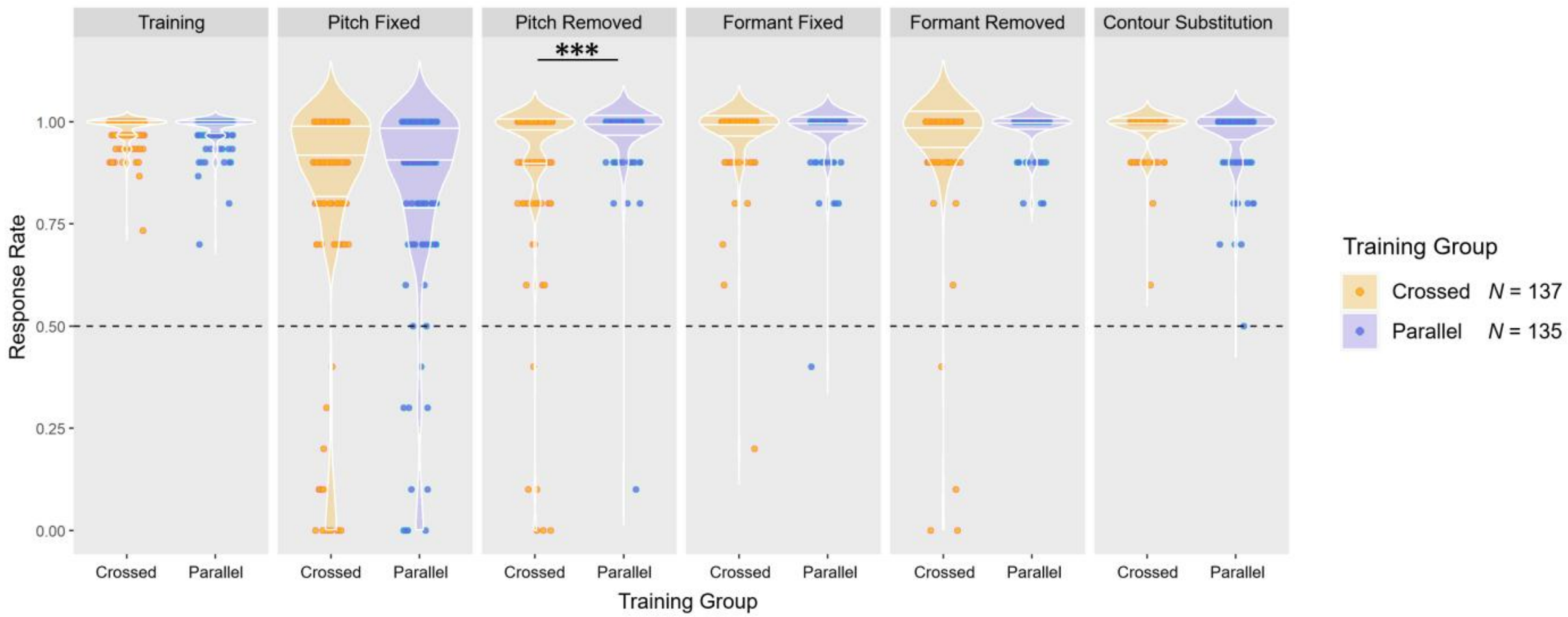

c

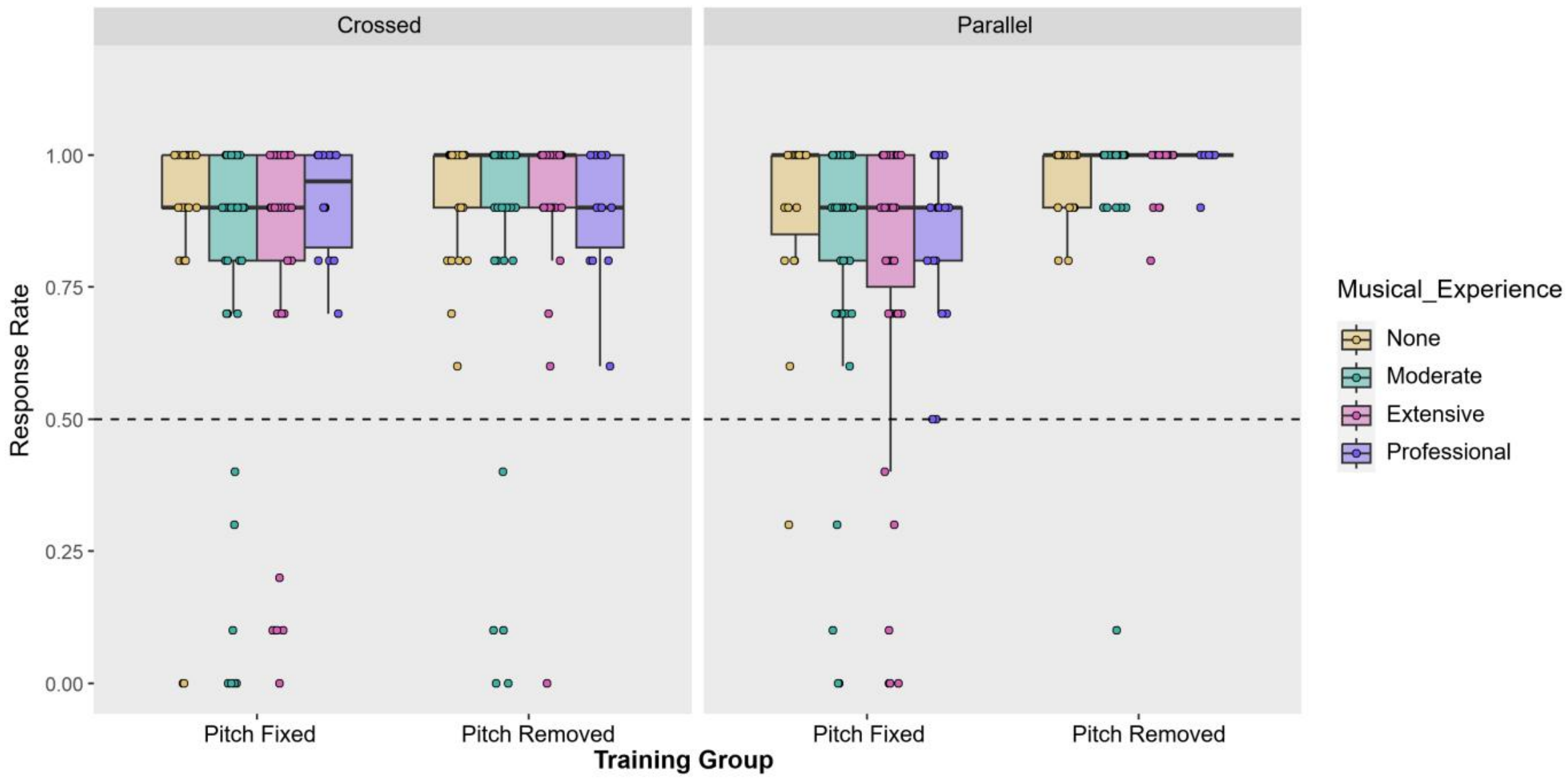

a, Comparison of Response rates between two training groups (Crossed-Contours, *N* = 11; Parallel-Contours, *N* = 12) in zebra finches experiment. **b,** Comparison of Response rates between two training groups (Crossed-Contours, *N* = 137; Parallel-Contours, *N* = 135) in human online task. **c,** Response rates of human participants with different levels of musical experience when responding to pitch-fixed and pitch-removed stimuli. Human participants were categorized according to musical experience as None (Crossed-Contours, *N* = 27; Parallel-Contours, *N* = 19), Moderate (Crossed-Contours, *N* = 51; Parallel-Contours, *N* = 56), Extensive (Crossed-Contours, *N* = 45; Parallel-Contours, *N* = 43), or Professional (Crossed-Contours, *N* = 14; Parallel-Contours, *N* = 17). Significant differences are indicated as follows: *** $P \leq 0.001$; non-indicated comparisons are not significant ($p > 0.05$). The violin plots in panels a and b show kernel density estimates, with white horizontal lines marking the median, first quartile, and third quartile. The box plots in panel c display the median and interquartile range, with whiskers extending to 1.5 times the interquartile range. The horizontal dashed line in all panels indicates the chance level (50%).

Fig. S2: Stimuli discrimination performance.

a

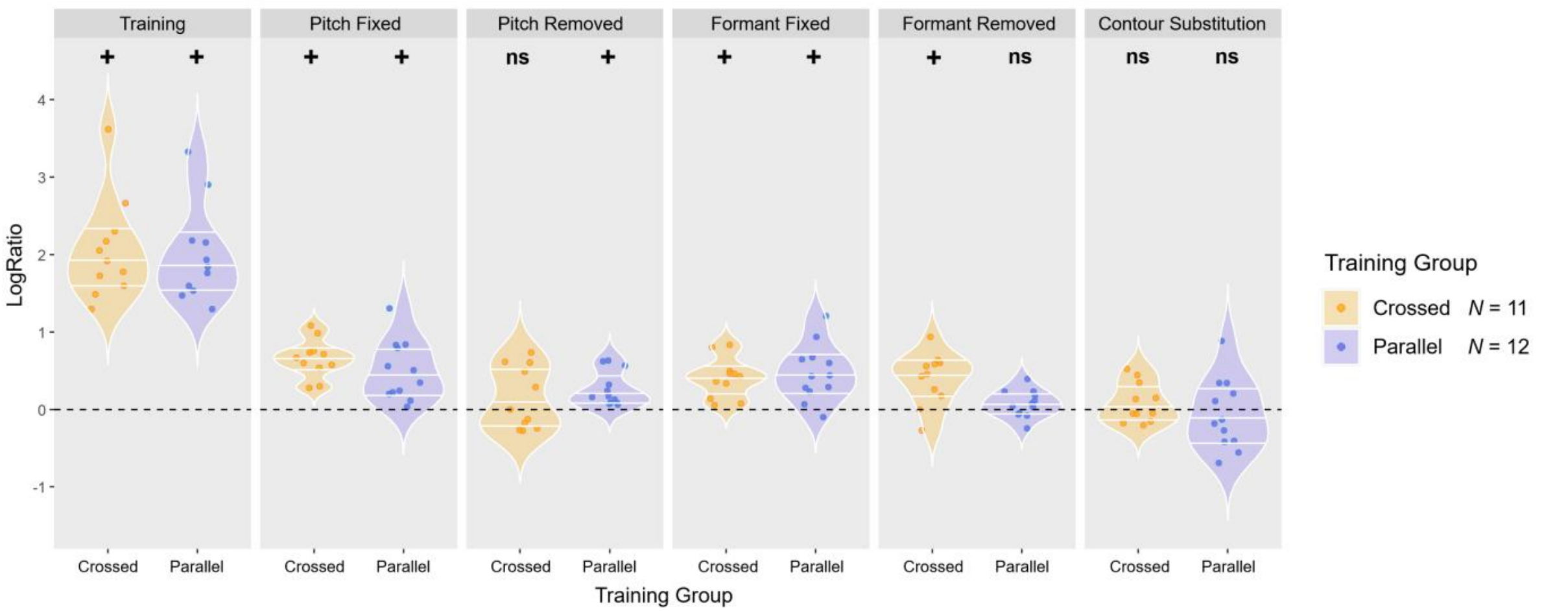

b

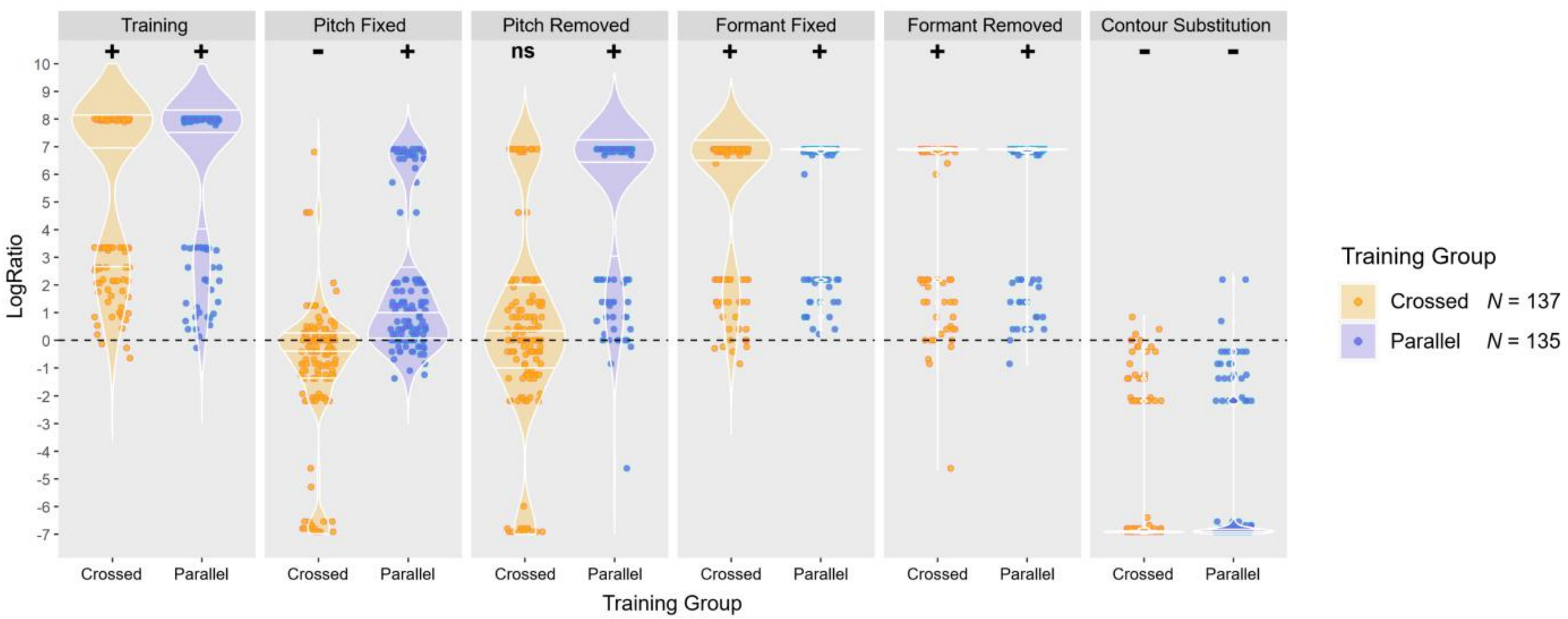

c

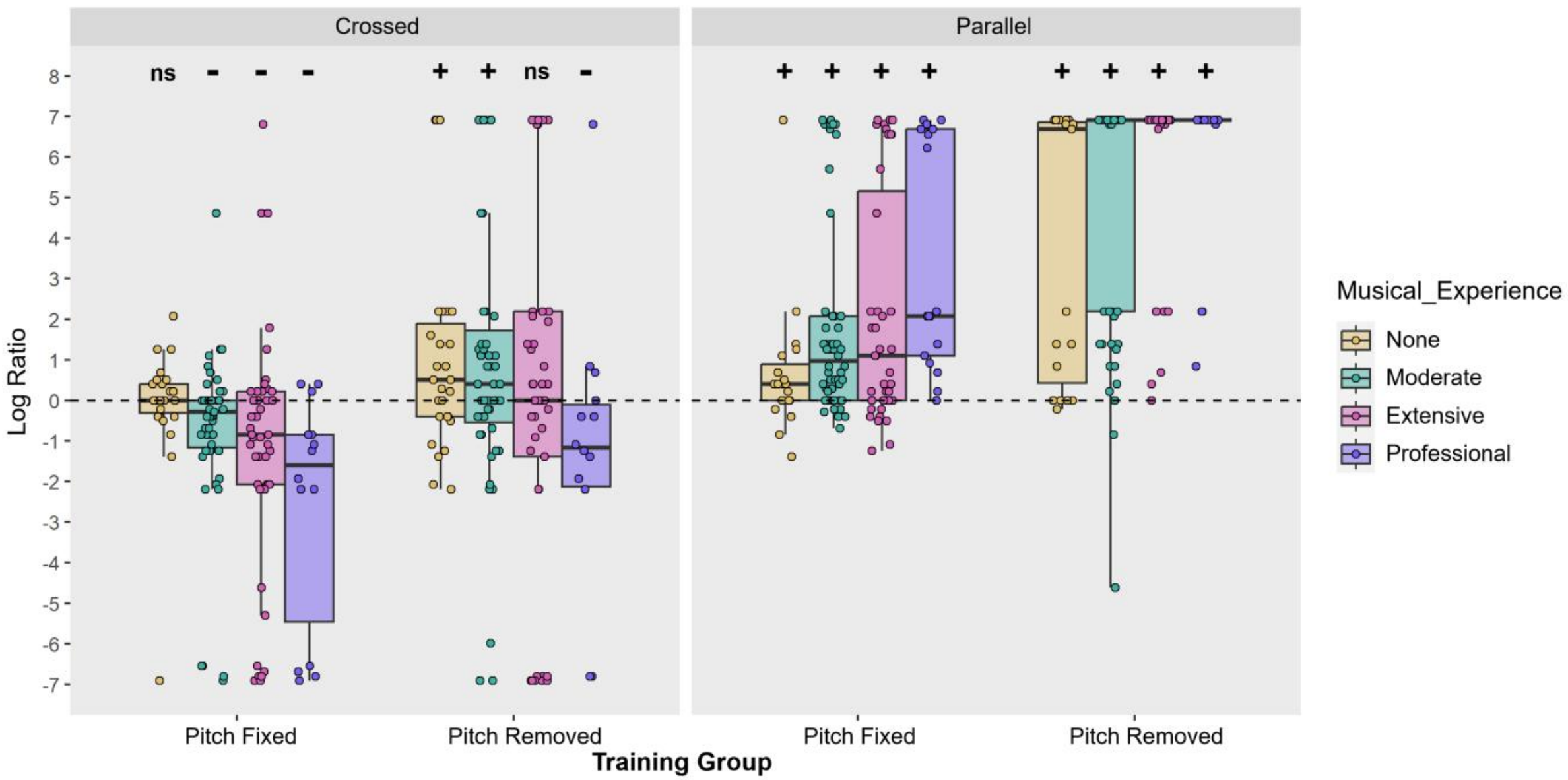

a, Visualization of the logRatios for two training groups (Crossed-Contours, *N* = 11; Parallel-Contours, *N* = 12) responding to the various test stimuli in zebra finches experiment. **b,** Visualization of the logRatios for two training groups (Crossed-Contours, *N* = 137; Parallel-Contours, *N* = 135) in human online task. **c,** logRatios of human participants with different levels of musical experience when responding to pitch-fixed and pitch-removed stimuli. Human participants were categorized according to musical experience as None (Crossed-Contours, *N* = 27; Parallel-Contours, *N* = 19), Moderate (Crossed-Contours, *N* = 51; Parallel-Contours, *N* = 56), Extensive (Crossed-Contours, *N* = 45; Parallel-Contours, *N* = 43), or Professional (Crossed-Contours, *N* = 14; Parallel-Contours, *N* = 17). A "+" indicates that the Log (similar/dissimilar) of a Test treatment is significantly greater than 0; a "–" indicates the LogRatio value significantly below 0; and a "ns" denotes that the LogRatio value does not differ significantly from 0. The violin plots in panels a and b represent kernel density estimations, with white horizontal lines indicating the median, first, and third quartiles. The box plots in panel c display the median and the first and third quartiles, with whiskers extending to 1.5 times the interquartile range. The Horizontal dashed line in panel a represents chance level, which was 50% for both tasks. In both panels, the dashed lines represent the discrimination boundaries, where the probability of classifying a test stimulus as similar to its corresponding training stimulus equals the probability of classifying it as similar to the opposite training stimulus.

Table S1 Summary of the GLMs selection.

Model	df	logLik	AICc	Δi	wi
Similarity scores[a] in zebra finch experiment					
1* Training_Group + Test_Stimuli + Test_Stimuli:Training_Group + (1|Bird_ID)	13	-408.694	846.3	0.00	0.299
2 Test_Stimuli + (1|Bird_ID)	7	-415.839	846.5	0.22	0.268
3 Training_Group + Test_Stimuli + (1|Bird_ID)	8	-415.021	847.2	0.84	0.197
4 Test_Stimuli + Sequence_Series + (1|Bird_ID)	8	-415.835	848.8	2.46	0.087
5 Training_Group + Test_Stimuli + Test_Stimuli:Training_Group + Sequence_Series + (1|Bird_ID)	14	-408.692	848.8	2.48	0.087
null (1|Bird_ID)	2	-764.165	1532.4	686.10	0.000
Response rate[b] of trials in zebra finch experiment					
1 Test_Stimuli + + (1|Bird_ID)	7	-446.703	908.3	0.00	0.363
2 Test_Stimuli + Sequence_Series + (1|Bird_ID)	8	-446.161	909.4	1.17	0.202
3 Training_Group + Test_Stimuli + (1|Bird_ID)	8	-446.210	909.5	1.27	0.192
4 Training_Group + Test_Stimuli + Sequence_Series + (1|Bird_ID)	9	-445.690	910.8	2.52	0.103
5* Training_Group + Test_Stimuli + Test_Stimuli:Training_Group + (1|Bird_ID)	13	-441.024	911.0	2.72	0.093
null (1|Bird_ID)	2	-715.047	1434.2	525.92	0.000
Similarity scores in human online task					
1* Training_Group + Test_Stimuli + Test_Stimuli:Training_Group + Musical_Experience + Test_Stimuli:Training_Group:Musical_Experience + (1|Participant_ID)	49	-2946.895	5994.9	0.00	0.790
2 Training_Group + Test_Stimuli + Test_Stimuli:Training_Group + Musical_Experience + Age_Stages + Test_Stimuli:Training_Group:Musical_Experience + (1|Participant_ID)	52	-2945.306	5998.1	3.22	0.158
3 Training_Group + Test_Stimuli + Test_Stimuli:Training_Group + Musical_Experience + Gender + Test_Stimuli:Training_Group:Musical_Experience + (1|Participant_ID)	52	-2946.623	6000.7	5.85	0.042
4 Training_Group + Test_Stimuli + Test_Stimuli:Training_Group + Musical_Experience + Age_Stages + Gender + Test_Stimuli:Training_Group:Musical_Experience + (1|Participant_ID)	55	-2944.950	6003.8	8.92	0.009
5 Training_Group + Test_Stimuli + Test_Stimuli:Training_Group + Musical_Experience + (1|Participant_ID)	16	-3046.884	6126.1	131.22	0.000
null (1|Participant_ID)	2	-7012.975	14030.0	8035.07	0.000
Response rate of trials in human online task					
1 Training_Group + Test_Stimuli + Test_Stimuli:Training_Group + Age_Stages + (1|Participant_ID)	16	-2478.004	4988.3	0.00	0.442
2 Training_Group + Test_Stimuli + Test_Stimuli:Training_Group + (1|Participant_ID)	13	-2482.129	4990.5	2.14	0.152
3 Training_Group + Test_Stimuli + Test_Stimuli:Training_Group + Age_Stages + Gender + (1|Participant_ID)	19	-2476.019	4990.5	2.16	0.150
4 Training_Group + Test_Stimuli + Test_Stimuli:Training_Group + Age_Stages + Musical_Experience + (1|Participant_ID)	19	-2476.582	4991.6	3.29	0.085
5 Training_Group + Test_Stimuli + Test_Stimuli:Training_Group + Gender + (1|Participant_ID)	16	-2479.770	4991.9	3.53	0.076

6*	Training_Group + Test_Stimuli + Test_Stimuli:Training_Group + Musical_Experience + Test_Stimuli:Training_Group:Musical_Experience + (1|Participant_ID)	49	-2468.409	5037.9	49.57	0.000

Best five models of the model selection (ranked by AICc and logLik), the chosen model, and the null models. The Akaike weight (wi) indicates the probability of a better model in the model candidates set, and Delta AICc (Δi) was used to show the difference in AICc score between the best model and the model being compared. A * indicates the model we choose.

a The proportion of response similar if human participants or zebra finches respond to one of two sounds.

b The proportion of trials that subjects respond with choosing A or B.

Table S2 Post hoc test results of Binomial GLMM for the interaction of Test_Stimuli & Training_Group in zebra finch experiment.

**Test_Stimuli**	**Training_Group**	**estimate**	**SE**	**z.ratio**	***p*.value**
Similarity score[a] of responses					
Training	Crossed - Parallel	-0.032	0.150	-0.216	0.8288
**Formant_Removed**	**Crossed - Parallel**	**0.347**	**0.114**	**3.040**	**0.0024**
Formant_Fixed	Crossed - Parallel	-0.081	0.112	-0.724	0.4690
Pitch_Fixed	Crossed - Parallel	0.148	0.113	1.305	0.1919
Pitch_Removed	Crossed - Parallel	-0.117	0.119	-0.986	0.3240
Contour_Substitution	Crossed - Parallel	0.128	0.111	1.155	0.2482
**Training - Formant_Removed**	**Crossed**	**1.522**	**0.129**	**11.806**	**<.0001**
**Training - Formant_Fixed**	**Crossed**	**1.544**	**0.128**	**12.109**	**<.0001**
**Training - Pitch_Fixed**	**Crossed**	**1.281**	**0.129**	**9.928**	**<.0001**
**Training - Pitch_Removed**	**Crossed**	**1.781**	**0.131**	**13.636**	**<.0001**
**Training - Contour_Substitution**	**Crossed**	**1.866**	**0.128**	**14.595**	**<.0001**
Formant_Removed - Formant_Fixed	Crossed	0.022	0.106	0.212	0.8324
**Formant_Removed - Pitch_Fixed**	**Crossed**	**-0.241**	**0.108**	**-2.233**	**0.0320**
**Formant_Removed - Pitch_Removed**	**Crossed**	**0.259**	**0.110**	**2.361**	**0.0248**
**Formant_Removed - Contour_Substitution**	**Crossed**	**0.344**	**0.106**	**3.236**	**0.0023**
**Formant_Fixed - Pitch_Fixed**	**Crossed**	**-0.263**	**0.106**	**-2.479**	**0.0198**
**Formant_Fixed - Pitch_Removed**	**Crossed**	**0.237**	**0.108**	**2.189**	**0.0330**
**Formant_Fixed - Contour_Substitution**	**Crossed**	**0.322**	**0.105**	**3.072**	**0.0035**
**Pitch_Fixed - Pitch_Removed**	**Crossed**	**0.500**	**0.110**	**4.550**	**<.0001**
**Pitch_Fixed - Contour_Substitution**	**Crossed**	**0.585**	**0.107**	**5.489**	**<.0001**
Pitch_Removed - Contour_Substitution	Crossed	0.085	0.108	0.786	0.4630
**Training - Formant_Removed**	**Parallel**	**1.901**	**0.127**	**14.937**	**<.0001**
**Training - Formant_Fixed**	**Parallel**	**1.496**	**0.126**	**11.824**	**<.0001**
**Training - Pitch_Fixed**	**Parallel**	**1.461**	**0.126**	**11.563**	**<.0001**
**Training - Pitch_Removed**	**Parallel**	**1.696**	**0.130**	**13.063**	**<.0001**
**Training - Contour_Substitution**	**Parallel**	**2.026**	**0.125**	**16.184**	**<.0001**
**Formant_Removed - Formant_Fixed**	**Parallel**	**-0.405**	**0.108**	**-3.765**	**0.0003**
**Formant_Removed - Pitch_Fixed**	**Parallel**	**-0.440**	**0.107**	**-4.093**	**0.0001**
Formant_Removed - Pitch_Removed	Parallel	-0.205	0.111	-1.839	0.0809
Formant_Removed - Contour_Substitution	Parallel	0.125	0.106	1.180	0.2549
Formant_Fixed - Pitch_Fixed	Parallel	-0.035	0.107	-0.325	0.7455
Formant_Fixed - Pitch_Removed	Parallel	0.200	0.111	1.811	0.0809
**Formant_Fixed - Contour_Substitution**	**Parallel**	**0.530**	**0.105**	**5.042**	**<.0001**
**Pitch_Fixed - Pitch_Removed**	**Parallel**	**0.235**	**0.110**	**2.127**	**0.0456**
**Pitch_Fixed - Contour_Substitution**	**Parallel**	**0.565**	**0.105**	**5.380**	**<.0001**
**Pitch_Removed - Contour_Substitution**	**Parallel**	**0.330**	**0.109**	**3.026**	**0.0037**
Response rate[b] of trials					
Training	Crossed - Parallel	-0.002	0.438	-0.005	0.9958

Formant_Removed	Crossed - Parallel	0.556	0.398	1.398	0.1620
Formant_Fixed	Crossed - Parallel	0.539	0.402	1.341	0.1801
Pitch_Fixed	Crossed - Parallel	0.449	0.402	1.115	0.2647
Pitch_Removed	Crossed - Parallel	0.352	0.394	0.893	0.3717
Contour_Substitution	Crossed - Parallel	0.148	0.399	0.369	0.7118
**Training - Formant_Removed**	**Crossed**	**1.590**	**0.187**	**8.509**	**<.0001**
**Training - Formant_Fixed**	**Crossed**	**1.202**	**0.193**	**6.238**	**<.0001**
**Training - Pitch_Fixed**	**Crossed**	**1.212**	**0.192**	**6.298**	**<.0001**
**Training - Pitch_Removed**	**Crossed**	**2.129**	**0.182**	**11.693**	**<.0001**
**Training - Contour_Substitution**	**Crossed**	**1.557**	**0.187**	**8.312**	**<.0001**
**Formant_Removed - Formant_Fixed**	**Crossed**	**-0.389**	**0.137**	**-2.844**	**0.0067**
**Formant_Removed - Pitch_Fixed**	**Crossed**	**-0.378**	**0.136**	**-2.773**	**0.0076**
**Formant_Removed - Pitch_Removed**	**Crossed**	**0.539**	**0.121**	**4.459**	**<.0001**
Formant_Removed - Contour_Substitution	Crossed	-0.033	0.129	-0.259	0.8525
Formant_Fixed - Pitch_Fixed	Crossed	0.010	0.144	0.072	0.9424
**Formant_Fixed - Pitch_Removed**	**Crossed**	**0.928**	**0.130**	**7.145**	**<.0001**
**Formant_Fixed - Contour_Substitution**	**Crossed**	**0.355**	**0.137**	**2.589**	**0.0120**
**Pitch_Fixed - Pitch_Removed**	**Crossed**	**0.917**	**0.130**	**7.080**	**<.0001**
**Pitch_Fixed - Contour_Substitution**	**Crossed**	**0.345**	**0.137**	**2.517**	**0.0136**
**Pitch_Removed - Contour_Substitution**	**Crossed**	**-0.572**	**0.122**	**-4.709**	**<.0001**
**Training - Formant_Removed**	**Parallel**	**2.149**	**0.164**	**13.140**	**<.0001**
**Training - Formant_Fixed**	**Parallel**	**1.743**	**0.166**	**10.516**	**<.0001**
**Training - Pitch_Fixed**	**Parallel**	**1.663**	**0.166**	**9.998**	**<.0001**
**Training - Pitch_Removed**	**Parallel**	**2.484**	**0.163**	**15.273**	**<.0001**
**Training - Contour_Substitution**	**Parallel**	**1.707**	**0.166**	**10.283**	**<.0001**
**Formant_Removed - Formant_Fixed**	**Parallel**	**-0.406**	**0.116**	**-3.508**	**0.0006**
**Formant_Removed - Pitch_Fixed**	**Parallel**	**-0.486**	**0.117**	**-4.162**	**0.0001**
**Formant_Removed - Pitch_Removed**	**Parallel**	**0.335**	**0.110**	**3.060**	**0.0028**
**Formant_Removed - Contour_Substitution**	**Parallel**	**-0.442**	**0.116**	**-3.805**	**0.0002**
Formant_Fixed - Pitch_Fixed	Parallel	-0.080	0.120	-0.664	0.5850
**Formant_Fixed - Pitch_Removed**	**Parallel**	**0.741**	**0.114**	**6.507**	**<.0001**
Formant_Fixed - Contour_Substitution	Parallel	-0.036	0.120	-0.300	0.7639
**Pitch_Fixed - Pitch_Removed**	**Parallel**	**0.821**	**0.115**	**7.140**	**<.0001**
Pitch_Fixed - Contour_Substitution	Parallel	0.044	0.121	0.363	0.7639
**Pitch_Removed - Contour_Substitution**	**Parallel**	**-0.777**	**0.114**	**-6.794**	**<.0001**

Response variables in GLMMs. “Crossed” refers to the Crossed_Contours training group, and “Parallel” refers to the Parallel_Contours training group. Statistical comparisons were performed using two-sided pairwise contrasts of estimated marginal means (EMMs) following the GLMMs. No adjustment for multiple comparisons was applied to the planned comparisons between the two training groups for each stimulus, whereas FDR correction was applied to comparisons among stimuli within each training group. Each of the pairwise multiple comparison tests is separated by borders within the table. Bold indicates significant differences <0.05.

a The proportion of response similar if birds respond to one of two sounds.

b The proportion of trials that birds respond with pecking sensor A or B.

Table S3 Post hoc test results of Binomial GLMM for the interaction of Test_Stimuli & Training_Group in human online task.

Test_Stimuli	Training_Group	estimate	SE	z.ratio	*p*.value
Similarity score[a] of responses					
Training	Crossed - Parallel	-0.116	0.084	-1.384	0.1663
Formant_Removed	Crossed - Parallel	-0.084	0.131	-0.642	0.5206
Formant_Fixed	Crossed - Parallel	-0.168	0.128	-1.309	0.1904
**Pitch_Fixed**	**Crossed - Parallel**	**-1.262**	**0.104**	**12.086**	**<.0001**
**Pitch_Removed**	**Crossed - Parallel**	**-1.784**	**0.113**	**15.806**	**<.0001**
Contour_Substitution	Crossed - Parallel	-0.061	0.128	-0.480	0.6312
Training - Formant_Removed	Crossed	-0.020	0.098	-0.206	0.8369
Training - Formant_Fixed	Crossed	0.041	0.095	0.435	0.7110
**Training - Pitch_Fixed**	**Crossed**	**2.358**	**0.080**	**29.440**	**<.0001**
**Training - Pitch_Removed**	**Crossed**	**1.856**	**0.077**	**23.961**	**<.0001**
**Training - Contour_Substitution**	**Crossed**	**3.765**	**0.098**	**38.613**	**<.0001**
Formant_Removed - Formant_Fixed	Crossed	0.0612	0.118	0.518	0.6971
**Formant_Removed - Pitch_Fixed**	**Crossed**	**2.378**	**0.107**	**22.238**	**<.0001**
**Formant_Removed - Pitch_Removed**	**Crossed**	**1.876**	**0.105**	**17.869**	**<.0001**
**Formant_Removed - Contour_Substitution**	**Crossed**	**3.785**	**0.121**	**31.396**	**<.0001**
**Formant_Fixed - Pitch_Fixed**	**Crossed**	**2.317**	**0.104**	**22.293**	**<.0001**
**Formant_Fixed - Pitch_Removed**	**Crossed**	**1.814**	**0.102**	**17.806**	**<.0001**
**Formant_Fixed - Contour_Substitution**	**Crossed**	**3.724**	**0.118**	**31.590**	**<.0001**
**Pitch_Fixed - Pitch_Removed**	**Crossed**	**-0.503**	**0.088**	**-5.714**	**<.0001**
**Pitch_Fixed - Contour_Substitution**	**Crossed**	**1.407**	**0.106**	**13.295**	**<.0001**
**Pitch_Removed - Contour_Substitution**	**Crossed**	**1.910**	**0.104**	**18.378**	**<.0001**
Training - Formant_Removed	Parallel	0.012	0.100	0.120	0.9157
Training - Formant_Fixed	Parallel	-0.011	0.100	-0.106	0.9157
**Training - Pitch_Fixed**	**Parallel**	**1.212**	**0.085**	**14.347**	**<.0001**
Training - Pitch_Removed	Parallel	0.187	0.097	1.939	0.0787
**Training - Contour_Substitution**	**Parallel**	**3.820**	**0.098**	**38.972**	**<.0001**
Formant_Removed - Formant_Fixed	Parallel	-0.023	0.123	-0.184	0.9157
**Formant_Removed - Pitch_Fixed**	**Parallel**	**1.200**	**0.110**	**10.896**	**<.0001**
Formant_Removed - Pitch_Removed	Parallel	0.175	0.120	1.465	0.1787
**Formant_Removed - Contour_Substitution**	**Parallel**	**3.808**	**0.121**	**31.515**	**<.0001**
**Formant_Fixed - Pitch_Fixed**	**Parallel**	**1.223**	**0.110**	**11.102**	**<.0001**
Formant_Fixed - Pitch_Removed	Parallel	0.198	0.120	1.654	0.1338
**Formant_Fixed - Contour_Substitution**	**Parallel**	**3.830**	**0.121**	**31.704**	**<.0001**
**Pitch_Fixed - Pitch_Removed**	**Parallel**	**-1.025**	**0.107**	**-9.577**	**<.0001**
**Pitch_Fixed - Contour_Substitution**	**Parallel**	**2.608**	**0.108**	**24.180**	**<.0001**
**Pitch_Removed - Contour_Substitution**	**Parallel**	**3.632**	**0.118**	**30.801**	**<.0001**
Response rate[b] of trials					
Training	Crossed - Parallel	0.003	0.092	0.037	0.9702

Formant_Removed	Crossed - Parallel	-0.179	0.140	-1.274	0.2026
Formant_Fixed	Crossed - Parallel	-0.033	0.142	-0.230	0.8181
Pitch_Fixed	Crossed - Parallel	0.038	0.112	0.339	0.7343
**Pitch_Removed**	**Crossed - Parallel**	**-0.484**	**0.131**	**-3.689**	**0.0002**
Contour_Substitution	Crossed - Parallel	0.160	0.142	1.120	0.2628
Training - Formant_Removed	Crossed	0.168	0.105	1.595	0.1589
Training - Formant_Fixed	Crossed	0.035	0.108	0.319	0.7498
**Training - Pitch_Fixed**	**Crossed**	**0.898**	**0.091**	**9.873**	**<.0001**
**Training - Pitch_Removed**	**Crossed**	**0.524**	**0.095**	**5.536**	**<.0001**
Training - Contour_Substitution	Crossed	-0.040	0.112	-0.362	0.7498
Formant_Removed - Formant_Fixed	Crossed	-0.133	0.130	-1.026	0.3809
**Formant_Removed - Pitch_Fixed**	**Crossed**	**0.730**	**0.116**	**6.313**	**<.0001**
**Formant_Removed - Pitch_Removed**	**Crossed**	**0.357**	**0.119**	**3.007**	**0.0044**
Formant_Removed - Contour_Substitution	Crossed	-0.208	0.133	-1.570	0.1589
**Formant_Fixed - Pitch_Fixed**	**Crossed**	**0.863**	**0.119**	**7.283**	**<.0001**
**Formant_Fixed - Pitch_Removed**	**Crossed**	**0.490**	**0.122**	**4.033**	**0.0001**
Formant_Fixed - Contour_Substitution	Crossed	-0.075	0.135	-0.555	0.6680
**Pitch_Fixed - Pitch_Removed**	**Crossed**	**-0.373**	**0.106**	**-3.510**	**0.0008**
**Pitch_Fixed - Contour_Substitution**	**Crossed**	**-0.938**	**0.122**	**-7.711**	**<.0001**
**Pitch_Removed - Contour_Substitution**	**Crossed**	**-0.565**	**0.125**	**-4.536**	**<.0001**
Training - Formant_Removed	Parallel	-0.014	0.111	-0.131	0.9884
Training - Formant_Fixed	Parallel	-0.002	0.110	-0.015	0.9884
**Training - Pitch_Fixed**	**Parallel**	**0.932**	**0.089**	**10.448**	**<.0001**
Training - Pitch_Removed	Parallel	0.038	0.109	0.345	0.9624
Training - Contour_Substitution	Parallel	0.116	0.107	1.083	0.6972
Formant_Removed - Formant_Fixed	Parallel	0.013	0.135	0.095	0.9884
**Formant_Removed - Pitch_Fixed**	**Parallel**	**0.947**	**0.119**	**7.967**	**<.0001**
Formant_Removed - Pitch_Removed	Parallel	0.052	0.134	0.387	0.9624
Formant_Removed - Contour_Substitution	Parallel	0.130	0.132	0.981	0.6996
**Formant_Fixed - Pitch_Fixed**	**Parallel**	**0.934**	**0.118**	**7.894**	**<.0001**
Formant_Fixed - Pitch_Removed	Parallel	0.039	0.134	0.292	0.9624
Formant_Fixed - Contour_Substitution	Parallel	0.117	0.132	0.887	0.7029
**Pitch_Fixed - Pitch_Removed**	**Parallel**	**-0.895**	**0.117**	**-7.640**	**<.0001**
**Pitch_Fixed - Contour_Substitution**	**Parallel**	**-0.817**	**0.115**	**-7.085**	**<.0001**
Pitch_Removed - Contour_Substitution	Parallel	0.078	0.131	0.596	0.9185

Response variables in GLMMs. "Crossed" refers to the Crossed_Contours training group, and "Parallel" refers to the Parallel_Contours training group. Statistical comparisons were performed using two-sided pairwise contrasts of estimated marginal means (EMMs) following the GLMMs. No adjustment for multiple comparisons was applied to the planned comparisons between the two training groups for each stimulus, whereas FDR correction was applied to comparisons among stimuli within each training group. Each of the pairwise multiple comparison tests is separated by borders within the table. Bold indicates significant differences <0.05.

a The proportion of response similar if participant respond to one of two sounds.

b The proportion of trials that participants respond with choosing option A or B.

Table S4 Post hoc test results of Binomial GLMM for the interaction of Musical_Experience, Test_Stimuli, and Training_Group in human online task.

Test_Stimuli	Training_Group	Musical_Experience	estimate	SE	z.ratio	*p*.value
Similarity score[a] of responses						
**Training**	**Crossed**	**None - Moderate**	**-0.347**	**0.136**	**-2.552**	**0.0214**
**Training**	**Crossed**	**None - Extensive**	**-0.717**	**0.145**	**-4.940**	**<.0001**
Training	Crossed	None - Professional	-0.375	0.193	-1.941	0.0783
**Training**	**Crossed**	**Moderate - Extensive**	**-0.370**	**0.129**	**-2.865**	**0.0125**
Training	Crossed	Moderate - Professional	-0.028	0.182	-0.156	0.8762
Training	Crossed	Extensive - Professional	0.342	0.189	1.813	0.0839
**Training**	**Parallel**	**None - Moderate**	**-0.581**	**0.153**	**-3.794**	**0.0004**
**Training**	**Parallel**	**None - Extensive**	**-0.617**	**0.161**	**-3.839**	**0.0004**
**Training**	**Parallel**	**None - Professional**	**-0.705**	**0.204**	**-3.459**	**0.0011**
Training	Parallel	Moderate - Extensive	-0.036	0.129	-0.274	0.7838
Training	Parallel	Moderate - Professional	-0.124	0.180	-0.687	0.7378
Training	Parallel	Extensive - Professional	-0.088	0.187	-0.474	0.7630
**Formant_Removed**	**Crossed**	**None - Moderate**	**-0.499**	**0.205**	**-2.436**	**0.0446**
**Formant_Removed**	**Crossed**	**None - Extensive**	**-0.784**	**0.218**	**-3.594**	**0.0020**
Formant_Removed	Crossed	None - Professional	-0.671	0.306	-2.196	0.0562
Formant_Removed	Crossed	Moderate - Extensive	-0.286	0.203	-1.410	0.2377
Formant_Removed	Crossed	Moderate - Professional	-0.173	0.295	-0.586	0.6692
Formant_Removed	Crossed	Extensive - Professional	0.113	0.304	0.371	0.7105
**Formant_Removed**	**Parallel**	**None - Moderate**	**-0.678**	**0.228**	**-2.968**	**0.0090**
**Formant_Removed**	**Parallel**	**None - Extensive**	**-0.728**	**0.241**	**-3.024**	**0.0090**
**Formant_Removed**	**Parallel**	**None - Professional**	**-0.817**	**0.312**	**-2.617**	**0.0178**
Formant_Removed	Parallel	Moderate - Extensive	-0.050	0.202	-0.250	0.8029
Formant_Removed	Parallel	Moderate - Professional	-0.139	0.283	-0.490	0.8029
Formant_Removed	Parallel	Extensive - Professional	-0.088	0.293	-0.302	0.8029
**Formant_Fixed**	**Crossed**	**None - Moderate**	**-0.530**	**0.202**	**-2.627**	**0.0259**
**Formant_Fixed**	**Crossed**	**None - Extensive**	**-0.850**	**0.217**	**-3.915**	**0.0005**
Formant_Fixed	Crossed	None - Professional	-0.520	0.290	-1.794	0.1456
Formant_Fixed	Crossed	Moderate - Extensive	-0.320	0.201	-1.595	0.1661
Formant_Fixed	Crossed	Moderate - Professional	0.010	0.278	0.037	0.9707
Formant_Fixed	Crossed	Extensive - Professional	0.330	0.289	1.143	0.3036
**Formant_Fixed**	**Parallel**	**None - Moderate**	**-0.648**	**0.233**	**-2.777**	**0.0251**
**Formant_Fixed**	**Parallel**	**None - Extensive**	**-0.643**	**0.244**	**-2.636**	**0.0251**
Formant_Fixed	Parallel	None - Professional	-0.676	0.311	-2.174	0.0593
Formant_Fixed	Parallel	Moderate - Extensive	0.005	0.204	0.024	0.9812
Formant_Fixed	Parallel	Moderate - Professional	-0.028	0.280	-0.099	0.9812
Formant_Fixed	Parallel	Extensive - Professional	-0.033	0.289	-0.112	0.9812
**Pitch_Fixed**	**Crossed**	**None - Moderate**	**0.414**	**0.172**	**2.411**	**0.0239**
**Pitch_Fixed**	**Crossed**	**None - Extensive**	**0.606**	**0.176**	**3.442**	**0.0017**

**Pitch_Fixed**	**Crossed**	**None - Professional**	**0.999**	**0.244**	**4.093**	**0.0003**
Pitch_Fixed	Crossed	Moderate - Extensive	0.192	0.152	1.260	0.2077
**Pitch_Fixed**	**Crossed**	**Moderate - Professional**	**0.585**	**0.228**	**2.570**	**0.0203**
Pitch_Fixed	Crossed	Extensive - Professional	0.393	0.231	1.703	0.1064
**Pitch_Fixed**	**Parallel**	**None - Moderate**	**-0.400**	**0.192**	**-2.085**	**0.0445**
**Pitch_Fixed**	**Parallel**	**None - Extensive**	**-0.427**	**0.201**	**-2.119**	**0.0445**
**Pitch_Fixed**	**Parallel**	**None - Professional**	**-0.968**	**0.260**	**-3.725**	**0.0012**
Pitch_Fixed	Parallel	Moderate - Extensive	-0.027	0.155	-0.174	0.8621
**Pitch_Fixed**	**Parallel**	**Moderate - Professional**	**-0.569**	**0.226**	**-2.518**	**0.0354**
**Pitch_Fixed**	**Parallel**	**Extensive - Professional**	**-0.542**	**0.234**	**-2.314**	**0.0413**
Pitch_Removed	Crossed	None - Moderate	0.106	0.168	0.634	0.5260
Pitch_Removed	Crossed	None - Extensive	0.238	0.170	1.405	0.2402
**Pitch_Removed**	**Crossed**	**None - Professional**	**0.966**	**0.236**	**4.091**	**0.0002**
Pitch_Removed	Crossed	Moderate - Extensive	0.132	0.143	0.921	0.4284
**Pitch_Removed**	**Crossed**	**Moderate - Professional**	**0.859**	**0.218**	**3.943**	**0.0002**
**Pitch_Removed**	**Crossed**	**Extensive - Professional**	**0.727**	**0.220**	**3.313**	**0.0018**
**Pitch_Removed**	**Parallel**	**None - Moderate**	**-0.524**	**0.216**	**-2.424**	**0.0307**
**Pitch_Removed**	**Parallel**	**None - Extensive**	**-0.861**	**0.234**	**-3.673**	**0.0014**
**Pitch_Removed**	**Parallel**	**None - Professional**	**-0.906**	**0.301**	**-3.008**	**0.0079**
Pitch_Removed	Parallel	Moderate - Extensive	-0.337	0.191	-1.763	0.1167
Pitch_Removed	Parallel	Moderate - Professional	-0.382	0.269	-1.422	0.1861
Pitch_Removed	Parallel	Extensive - Professional	-0.045	0.284	-0.160	0.8728
Contour_Substitution	Crossed	None - Moderate	0.337	0.204	1.653	0.1969
Contour_Substitution	Crossed	None - Extensive	0.558	0.215	2.589	0.0578
Contour_Substitution	Crossed	None - Professional	0.611	0.307	1.994	0.1386
Contour_Substitution	Crossed	Moderate - Extensive	0.221	0.193	1.143	0.3797
Contour_Substitution	Crossed	Moderate - Professional	0.274	0.291	0.941	0.4159
Contour_Substitution	Crossed	Extensive - Professional	0.053	0.300	0.178	0.8586
**Contour_Substitution**	**Parallel**	**None - Moderate**	**1.022**	**0.216**	**4.730**	**<.0001**
**Contour_Substitution**	**Parallel**	**None - Extensive**	**0.982**	**0.226**	**4.341**	**<.0001**
**Contour_Substitution**	**Parallel**	**None - Professional**	**1.125**	**0.301**	**3.742**	**0.0004**
Contour_Substitution	Parallel	Moderate - Extensive	-0.040	0.197	-0.203	0.8392
Contour_Substitution	Parallel	Moderate - Professional	0.103	0.279	0.367	0.8392
Contour_Substitution	Parallel	Extensive - Professional	0.143	0.287	0.497	0.8392
Response rate[b] of trials						
Training	Crossed	None - Moderate	-0.033	0.157	-0.209	0.8825
Training	Crossed	None - Extensive	-0.098	0.161	-0.604	0.8825
Training	Crossed	None - Professional	-0.067	0.219	-0.307	0.8825
Training	Crossed	Moderate - Extensive	-0.065	0.137	-0.475	0.8825
Training	Crossed	Moderate - Professional	-0.035	0.201	-0.171	0.8825
Training	Crossed	Extensive - Professional	0.030	0.205	0.148	0.8825
Training	Parallel	None - Moderate	0.003	0.176	0.016	0.9933
Training	Parallel	None - Extensive	0.004	0.182	0.022	0.9933

Training	Parallel	None - Professional	-0.062	0.223	-0.277	0.9933
Training	Parallel	Moderate - Extensive	0.001	0.134	0.008	0.9933
Training	Parallel	Moderate - Professional	-0.065	0.186	-0.348	0.9933
Training	Parallel	Extensive - Professional	-0.066	0.192	-0.343	0.9933
Formant_Removed	Crossed	None - Moderate	0.382	0.236	1.618	0.6107
Formant_Removed	Crossed	None - Extensive	0.134	0.247	0.545	0.8786
Formant_Removed	Crossed	None - Professional	0.100	0.332	0.302	0.9105
Formant_Removed	Crossed	Moderate - Extensive	-0.247	0.194	-1.271	0.6107
Formant_Removed	Crossed	Moderate - Professional	-0.281	0.296	-0.952	0.6821
Formant_Removed	Crossed	Extensive - Professional	-0.034	0.304	-0.112	0.9105
Formant_Removed	Parallel	None - Moderate	0.064	0.275	0.234	0.9634
Formant_Removed	Parallel	None - Extensive	-0.014	0.287	-0.050	0.9634
Formant_Removed	Parallel	None - Professional	-0.028	0.350	-0.081	0.9634
Formant_Removed	Parallel	Moderate - Extensive	-0.079	0.210	-0.376	0.9634
Formant_Removed	Parallel	Moderate - Professional	-0.093	0.289	-0.320	0.9634
Formant_Removed	Parallel	Extensive - Professional	-0.014	0.301	-0.046	0.9634
Formant_Fixed	Crossed	None - Moderate	0.006	0.239	0.024	0.9841
Formant_Fixed	Crossed	None - Extensive	-0.093	0.248	-0.376	0.9841
Formant_Fixed	Crossed	None - Professional	-0.100	0.340	-0.293	0.9841
Formant_Fixed	Crossed	Moderate - Extensive	-0.099	0.209	-0.475	0.9841
Formant_Fixed	Crossed	Moderate - Professional	-0.105	0.312	-0.338	0.9841
Formant_Fixed	Crossed	Extensive - Professional	-0.006	0.319	-0.020	0.9841
Formant_Fixed	Parallel	None - Moderate	0.013	0.271	0.046	0.9635
Formant_Fixed	Parallel	None - Extensive	-0.066	0.284	-0.232	0.9635
Formant_Fixed	Parallel	None - Professional	-0.080	0.347	-0.230	0.9635
Formant_Fixed	Parallel	Moderate - Extensive	-0.078	0.210	-0.374	0.9635
Formant_Fixed	Parallel	Moderate - Professional	-0.092	0.289	-0.318	0.9635
Formant_Fixed	Parallel	Extensive - Professional	-0.014	0.301	-0.046	0.9635
Pitch_Fixed	Crossed	None - Moderate	0.366	0.189	1.934	0.1062
Pitch_Fixed	Crossed	None - Extensive	0.249	0.194	1.279	0.3014
Pitch_Fixed	Crossed	None - Professional	-0.272	0.281	-0.969	0.3990
Pitch_Fixed	Crossed	Moderate - Extensive	-0.118	0.157	-0.750	0.4531
Pitch_Fixed	Crossed	Moderate - Professional	-0.639	0.257	-2.489	0.0769
Pitch_Fixed	Crossed	Extensive - Professional	-0.521	0.260	-2.001	0.1062
Pitch_Fixed	Parallel	None - Moderate	0.271	0.221	1.222	0.3430
Pitch_Fixed	Parallel	None - Extensive	0.540	0.226	2.395	0.0997
Pitch_Fixed	Parallel	None - Professional	0.327	0.272	1.204	0.3430
Pitch_Fixed	Parallel	Moderate - Extensive	0.270	0.156	1.728	0.2522
Pitch_Fixed	Parallel	Moderate - Professional	0.057	0.218	0.260	0.7949
Pitch_Fixed	Parallel	Extensive - Professional	-0.213	0.222	-0.961	0.4036
Pitch_Removed	Crossed	None - Moderate	0.305	0.211	1.446	0.4444
Pitch_Removed	Crossed	None - Extensive	-0.047	0.222	-0.211	0.8331
Pitch_Removed	Crossed	None - Professional	0.225	0.287	0.784	0.6495

Pitch_Removed	Crossed	Moderate - Extensive	-0.351	0.181	-1.944	0.3112
Pitch_Removed	Crossed	Moderate - Professional	-0.080	0.256	-0.310	0.8331
Pitch_Removed	Crossed	Extensive - Professional	0.272	0.266	1.022	0.6132
Pitch_Removed	Parallel	None - Moderate	-0.194	0.256	-0.756	0.6904
Pitch_Removed	Parallel	None - Extensive	-0.312	0.271	-1.151	0.6904
Pitch_Removed	Parallel	None - Professional	-0.362	0.340	-1.065	0.6904
Pitch_Removed	Parallel	Moderate - Extensive	-0.118	0.210	-0.560	0.6904
Pitch_Removed	Parallel	Moderate - Professional	-0.168	0.294	-0.572	0.6904
Pitch_Removed	Parallel	Extensive - Professional	-0.050	0.307	-0.165	0.8693
Contour_Substitution	Crossed	None - Moderate	0.039	0.247	0.158	0.9711
Contour_Substitution	Crossed	None - Extensive	0.031	0.253	0.124	0.9711
Contour_Substitution	Crossed	None - Professional	-0.143	0.356	-0.401	0.9711
Contour_Substitution	Crossed	Moderate - Extensive	-0.008	0.210	-0.036	0.9711
Contour_Substitution	Crossed	Moderate - Professional	-0.182	0.327	-0.555	0.9711
Contour_Substitution	Crossed	Extensive - Professional	-0.174	0.332	-0.525	0.9711
Contour_Substitution	Parallel	None - Moderate	0.217	0.271	0.800	0.9061
Contour_Substitution	Parallel	None - Extensive	0.134	0.283	0.475	0.9061
Contour_Substitution	Parallel	None - Professional	0.185	0.337	0.551	0.9061
Contour_Substitution	Parallel	Moderate - Extensive	-0.083	0.199	-0.415	0.9061
Contour_Substitution	Parallel	Moderate - Professional	-0.032	0.270	-0.118	0.9061
Contour_Substitution	Parallel	Extensive - Professional	0.051	0.282	0.181	0.9061

Response variables in GLMMs. “Crossed” refers to the Crossed_Contours training group, and “Parallel” refers to the Parallel_Contours training group. Statistical comparisons were performed using two-sided pairwise contrasts of estimated marginal means (EMMs) following the GLMMs, with false discovery rate (FDR) correction for multiple comparisons. Each of the corrected pairwise multiple comparison tests is separated by borders within the table. Bold indicates significant differences <0.05.

a The proportion of response similar if participant respond to one of two sounds.

b The proportion of trials that participants respond with choosing option A or B.

Table S5 Estimates and 95% confidence intervals for the correct identification of test stimuli in zebra finch experiment.

Training_Group	Test_Stimuli	Emmean	SE	CL (95%)	
				Lower	Upper
Log(Cor/Inco) ~ Training_Group + Test_Stimuli + Test_Stimuli:Training_Group + (1|Bird_ID)					
Crossed	Training	1.9375	0.1072	1.7275	2.1476
Crossed	Formant_Removed	0.4157	0.0805	0.2579	0.5735
Crossed	Formant_Fixed	0.3934	0.0783	0.2399	0.5469
Crossed	Pitch_Fixed	0.6566	0.0807	0.4983	0.8148
Crossed	Pitch_Removed	0.1566	0.0832	-0.0065	0.3197
Crossed	Contour_Substitution	0.0713	0.0788	-0.0832	0.2258
Parallel	Training	1.9696	0.1044	1.7649	2.1743
Parallel	Formant_Removed	0.0690	0.0809	-0.0895	0.2275
Parallel	Formant_Fixed	0.4739	0.0796	0.3178	0.6300
Parallel	Pitch_Fixed	0.5088	0.0794	0.3531	0.6644
Parallel	Pitch_Removed	0.2737	0.0848	0.1075	0.4399
Parallel	Contour_Substitution	-0.0564	0.0775	-0.2083	0.0955

Lower CL and Upper CL represent the lower and upper 95% confidence limits (CL) of the confidence interval. "Crossed" refers to the Crossed_Contours training group, and "Parallel" refers to the Parallel_Contours training group. If zero is part of that confidence interval, the treatment combination Training_Group and Stimuli is not significantly different from 0. If both confidence levels are positive, then there is a bias toward response similar. If they are both negative, then they are biased toward response dissimilar.

Table S6 Estimates and 95% confidence intervals for the correct identification of test stimuli in human online task.

Training_Group	Test_Stimuli	Musical_Experience	Emmean	SE	CL (95%)	
					Lower	Upper
Log(Cor/Inco) ~ Training_Group + Test_Stimuli + Test_Stimuli:Training_Group + Musical_Experience + Test_Stimuli:Training_Group:Musical_Experience + (1|Participant_ID)						
Crossed	Training		1.8843	0.0583	1.770	1.999
Crossed	Formant_Removed		1.9044	0.0918	1.725	2.084
Crossed	Formant_Fixed		1.8432	0.0882	1.670	2.016
Crossed	Pitch_Fixed		-0.4740	0.0720	-0.615	-0.333
Crossed	Pitch_Removed		0.0288	0.0691	-0.107	0.164
Crossed	Contour_Substitution		-1.8806	0.0907	-2.058	-1.703
Parallel	Training		2.0003	0.0605	1.882	2.119
Parallel	Formant_Removed		1.9883	0.0930	1.806	2.171
Parallel	Formant_Fixed		2.0109	0.0930	1.829	2.193
Parallel	Pitch_Fixed		0.7883	0.0757	0.640	0.937
Parallel	Pitch_Removed		1.8129	0.0893	1.638	1.988
Parallel	Contour_Substitution		-1.8194	0.0899	-1.996	-1.643
Crossed	Training	None	1.524	0.1070	1.3145	1.734
Crossed	Training	Moderate	1.871	0.0840	1.7067	2.036
Crossed	Training	Extensive	2.242	0.0983	2.0491	2.434
Crossed	Training	Professional	1.900	0.1612	1.5838	2.216
Crossed	Formant_Removed	None	1.416	0.1557	1.1107	1.721
Crossed	Formant_Removed	Moderate	1.914	0.1330	1.6537	2.175
Crossed	Formant_Removed	Extensive	2.200	0.1531	1.9003	2.500
Crossed	Formant_Removed	Professional	2.087	0.2632	1.5714	2.603
Crossed	Formant_Fixed	None	1.368	0.1544	1.0655	1.671
Crossed	Formant_Fixed	Moderate	1.898	0.1302	1.6431	2.153
Crossed	Formant_Fixed	Extensive	2.218	0.1529	1.9188	2.518
Crossed	Formant_Fixed	Professional	1.888	0.2454	1.4071	2.369
Crossed	Pitch_Fixed	None	0.031	0.1367	-0.2368	0.299
Crossed	Pitch_Fixed	Moderate	-0.383	0.1042	-0.5876	-0.179
Crossed	Pitch_Fixed	Extensive	-0.575	0.1112	-0.7932	-0.357
Crossed	Pitch_Fixed	Professional	-0.968	0.2024	-1.3650	-0.572
Crossed	Pitch_Removed	None	0.356	0.1348	0.0923	0.621
Crossed	Pitch_Removed	Moderate	0.250	0.0997	0.0548	0.446
Crossed	Pitch_Removed	Extensive	0.118	0.1031	-0.0841	0.320
Crossed	Pitch_Removed	Professional	-0.609	0.1938	-0.9892	-0.229
Crossed	Contour_Substitution	None	-1.504	0.1590	-1.8160	-1.193
Crossed	Contour_Substitution	Moderate	-1.841	0.1275	-2.0910	-1.591
Crossed	Contour_Substitution	Extensive	-2.062	0.1454	-2.3469	-1.777
Crossed	Contour_Substitution	Professional	-2.115	0.2621	-2.6290	-1.602
Parallel	Training	None	1.525	0.1276	1.2745	1.775

Parallel	Training	Moderate	2.106	0.0850	1.9392	2.272
Parallel	Training	Extensive	2.141	0.0978	1.9496	2.333
Parallel	Training	Professional	2.230	0.1590	1.9179	2.541
Parallel	Formant_Removed	None	1.433	0.1864	1.0674	1.798
Parallel	Formant_Removed	Moderate	2.110	0.1322	1.8514	2.370
Parallel	Formant_Removed	Extensive	2.161	0.1527	1.8616	2.460
Parallel	Formant_Removed	Professional	2.249	0.2504	1.7584	2.740
Parallel	Formant_Fixed	None	1.519	0.1905	1.1457	1.892
Parallel	Formant_Fixed	Moderate	2.167	0.1350	1.9027	2.432
Parallel	Formant_Fixed	Extensive	2.162	0.1527	1.8631	2.462
Parallel	Formant_Fixed	Professional	2.195	0.2457	1.7134	2.677
Parallel	Pitch_Fixed	None	0.340	0.1634	0.0193	0.660
Parallel	Pitch_Fixed	Moderate	0.739	0.1004	0.5426	0.936
Parallel	Pitch_Fixed	Extensive	0.766	0.1178	0.5355	0.997
Parallel	Pitch_Fixed	Professional	1.308	0.2023	0.9115	1.705
Parallel	Pitch_Removed	None	1.240	0.1805	0.8865	1.594
Parallel	Pitch_Removed	Moderate	1.764	0.1190	1.5309	1.997
Parallel	Pitch_Removed	Extensive	2.101	0.1496	1.8078	2.394
Parallel	Pitch_Removed	Professional	2.146	0.2413	1.6736	2.619
Parallel	Contour_Substitution	None	-1.037	0.1717	-1.3738	-0.701
Parallel	Contour_Substitution	Moderate	-2.059	0.1313	-2.3166	-1.802
Parallel	Contour_Substitution	Extensive	-2.019	0.1474	-2.3080	-1.730
Parallel	Contour_Substitution	Professional	-2.162	0.2467	-2.6454	-1.678

Lower CL and Upper CL represent the lower and upper 95% confidence limits (CL) of the confidence interval. "Crossed" refers to the Crossed_Contours training group, and "Parallel" refers to the Parallel_Contours training group. If zero is part of that confidence interval, the treatment combination Training_Group and Stimuli is not significantly different from 0. If both confidence levels are positive, then there is a bias toward response similar. If they are both negative, then they are biased toward response dissimilar.
