# Supplementary material for: Divergent processing strategies for auditory sequence recognition in humans and zebra finches (Taeniopygia guttata)
Source: Commun Biol. 2026 Oct 13;9:1281. doi: 10.1038/s42003-026-11035-9 (PMC13661893; doi:10.1038/s42003-026-11035-9)
Supplement: Supplementary file 3 [file 42003_2026_11035_MOESM3_ESM.pdf]

Description of Additional Supplementary Files

Divergent Processing Strategies for Auditory Sequence Recognition in Humans and Zebra Finches (*Taeniopygia guttata*)

Supplementary Information: Supplementary Figures S1 and S2 and Supplementary Tables S1–S6 are provided together as a single Supplementary Information PDF.

File	Format	Description
Supplementary Information	PDF	Contains Supplementary Figures S1 and S2 and Supplementary Tables S1–S6.
Supplementary Data 1	XLSX	Source data for Figure 3, including raw behavioural scores from the 23 zebra finches included in the final experiment. The data also provide source data for Supplementary Figures S1a and S2a.
Supplementary Data 2	XLSX	Source data for Figure 4, including raw behavioural scores from the 272 human participants who completed the online listening categorization task. The data also provide source data for Supplementary Figures S1b,c and S2b,c.

Additional material availability: All stimulus materials generated in this study are publicly available via the Open Science Framework (OSF): https://doi.org/10.17605/OSF.IO/ZXDBJ59

The Supplementary Information PDF and the two Supplementary Data files are described here for clarity of the submission file inventory.
