# Supplementary material for: Divergent processing strategies for auditory sequence recognition in humans and zebra finches (Taeniopygia guttata)
Source: Commun Biol. 2026 Oct 13;9:1281. doi: 10.1038/s42003-026-11035-9 (PMC13661893; doi:10.1038/s42003-026-11035-9)
Supplement: Supplementary file 4 [file 42003_2026_11035_MOESM6_ESM.pdf]

nature portfolio

Corresponding author(s): Zhi-Yuan Ning; Henkjan Honing

Last updated by author(s): 2026/05/13

Reporting Summary

Nature Portfolio wishes to improve the reproducibility of the work that we publish. This form provides structure for consistency and transparency in reporting. For further information on Nature Portfolio policies, see our Editorial Policies and the Editorial Policy Checklist.

Statistics

For all statistical analyses, confirm that the following items are present in the figure legend, table legend, main text, or Methods section.

n/a	Confirmed	
☐	☒	The exact sample size (*n*) for each experimental group/condition, given as a discrete number and unit of measurement
☐	☒	A statement on whether measurements were taken from distinct samples or whether the same sample was measured repeatedly
☐	☒	The statistical test(s) used AND whether they are one- or two-sided<br>*Only common tests should be described solely by name; describe more complex techniques in the Methods section.*
☐	☒	A description of all covariates tested
☐	☒	A description of any assumptions or corrections, such as tests of normality and adjustment for multiple comparisons
☐	☒	A full description of the statistical parameters including central tendency (e.g. means) or other basic estimates (e.g. regression coefficient) AND variation (e.g. standard deviation) or associated estimates of uncertainty (e.g. confidence intervals)
☐	☒	For null hypothesis testing, the test statistic (e.g. *F*, *t*, *r*) with confidence intervals, effect sizes, degrees of freedom and *P* value noted<br>*Give P values as exact values whenever suitable.*
☒	☐	For Bayesian analysis, information on the choice of priors and Markov chain Monte Carlo settings
☒	☐	For hierarchical and complex designs, identification of the appropriate level for tests and full reporting of outcomes
☒	☐	Estimates of effect sizes (e.g. Cohen's *d*, Pearson's *r*), indicating how they were calculated

Our web collection on statistics for biologists contains articles on many of the points above.

Software and code

Policy information about availability of computer code

Data collection	Data collection of the online listening categorization task involving human participants was fully automated, documenting stimulus codes, response choices, and reaction times (interval between stimulus onset and button click) directly into .csv files.<br>Likewise, data collection was also fully automated via computer-controlled operant chambers for birds.
Data analysis	Rstudio (R version 4.1.2)<br>The codes that support this study are available from the lead contact (Zhi-Yuan Ning; by email at zhiyuanning@hotmail.com) upon resonable request and are limited to non-commercial use.

For manuscripts utilizing custom algorithms or software that are central to the research but not yet described in published literature, software must be made available to editors and reviewers. We strongly encourage code deposition in a community repository (e.g. GitHub). See the Nature Portfolio guidelines for submitting code & software for further information.

Data

Policy information about availability of data

All manuscripts must include a data availability statement. This statement should provide the following information, where applicable:

- Accession codes, unique identifiers, or web links for publicly available datasets
- A description of any restrictions on data availability
- For clinical datasets or third party data, please ensure that the statement adheres to our policy

All data are included in the source data associated with this manuscript. Any additional information required to reanalyze the data reported in this paper is available from the lead contact (Zhi-Yuan Ning; by email at zhiyuanning@hotmail.com) upon resonable request and are limited to non-commercial use.

Research involving human participants, their data, or biological material

Policy information about studies with human participants or human data. See also policy information about sex, gender (identity/presentation), and sexual orientation and race, ethnicity and racism.

Reporting on sex and gender	Gender was self-reported by participants, coded as Male (M), Female (F), Other (X), or Undisclosed (U). Gender were considered as potential covariates during the initial analysis of auditory sequence recognition strategies; however, as no significant main effects or interactions were found, these variables were collapsed in the final models to prioritize parsimony.
Reporting on race, ethnicity, or other socially relevant groupings	Data on race and ethnicity were not collected, and no specific hypotheses regarding race or ethnicity were tested.
Population characteristics	The human study population (N = 272) spanned a wide age range, categorized into four stages: Early Adulthood (18–29 years old), Early Middle Age (30–44 years old), Late Middle Age (45–59 years old), and Seniorhood (60+ years old). These categories were used to evaluate whether auditory sequence recognition strategies shift with age. Analysis confirmed that the core findings regarding cue weighting were largely consistent across all age groups, with no significant main effect or interactions involving age detected. These variables were excluded in the final models.
Recruitment	Participants were recruited from students at the University of Amsterdam, Leiden University, and social media platforms. Participation was on an entirely voluntary basis, and no compensation was provided. All participants (ages 19–80 years; median age = 34 years) reported normal hearing. Recruitment focused on a broad range of musical and linguistic backgrounds to ensure a representative distribution of auditory cognitive abilities.
Ethics oversight	All procedures for the online listening categorization task involving human participants were approved by the Ethics Committee at the University of Amsterdam.

Note that full information on the approval of the study protocol must also be provided in the manuscript.

Field-specific reporting

Please select the one below that is the best fit for your research. If you are not sure, read the appropriate sections before making your selection.

☒ Life sciences ☐ Behavioural & social sciences ☐ Ecological, evolutionary & environmental sciences

For a reference copy of the document with all sections, see nature.com/documents/nr-reporting-summary-flat.pdf

Life sciences study design

All studies must disclose on these points even when the disclosure is negative.

Sample size	No statistical methods were used to predetermine sample size. Sample sizes were chosen based on previous experience with each assay.
Data exclusions	Songbirds were excluded from the final analysis if they failed to meet the pre-defined training criterion (e.g., >75% accuracy over three consecutive sessions). For human participants, data were excluded based on two pre-registered criteria: 1) failure to pass training phase, and 2) incomplete test sessions. Only subjects matching with these criteria were reported in the paper.
Replication	Experimental findings in both humans and birds were replicated across multiple individuals and subgroups (Sequence series) using identical operant protocols, there were no issues with replication.
Randomization	Stimulus presentation order was randomized for each session using a computer-generated pseudo-random sequence to prevent order effects. For the operant tasks, the assignment of specific sensor associated with specific stimulus was counterbalanced across subjects to control for potential bias toward sensor locations.
Blinding	Blinding was not applicable to the experimental setup as all data collection was fully automated via computer-controlled operant chambers (for birds) and an online interface (for humans).

Reporting for specific materials, systems and methods

We require information from authors about some types of materials, experimental systems and methods used in many studies. Here, indicate whether each material, system or method listed is relevant to your study. If you are not sure if a list item applies to your research, read the appropriate section before selecting a response.

Materials & experimental systems

n/a	Involved in the study
☒	☐ Antibodies
☒	☐ Eukaryotic cell lines
☒	☐ Palaeontology and archaeology
☐	☒ Animals and other organisms
☒	☐ Clinical data
☒	☐ Dual use research of concern
☒	☐ Plants

Methods

n/a	Involved in the study
☒	☐ ChIP-seq
☒	☐ Flow cytometry
☒	☐ MRI-based neuroimaging

Animals and other research organisms

Policy information about studies involving animals; ARRIVE guidelines recommended for reporting animal research, and Sex and Gender in Research

Laboratory animals	Twenty-three zebra finches (12 males and 11 females) completed the experiment. They were trained and tested at the age of 334 ± 47 days post-hatching (dph), (age males: M= 366, SD= 37, age females: M= 299, SD= 26). All birds originated from the in-house breeding colony at Leiden University. Birds lived in single-sex aviaries and transferred to standard laboratory cages (two same-sex birds per cage) for one-week acclimation prior to the experiment. None of 23 birds had any experience with this experimental setup or the stimuli preceding the experiment. Each experimental bird underwent a physical examination before being transferred to the Skinnerboxes. During the experiment, the health and welfare of these birds was monitored daily. If a bird did not obtain food by not using the sensors for 18 hours (a very rare event), the food hatch opened automatically.
Wild animals	The study did not involve wild animals.
Reporting on sex	Before we decide what kind of factors were suitable be included in the model analysis, we had already explored whether “Sex” could be set as one of those fix factors. The outcome suggested that “Sex” had almost negligible impact in training group (and subgroup) level, and that both sexes perform in the same pattern when tested with various novel stimuli. Furthermore, given the relatively small sample in this experiment, it would strongly reduce the statistical power if the “Sex” factor was introduced into a subgroup. Therefore, we decided not to put “Sex” as a factor in our final model analysis.
Field-collected samples	The study did not involve samples collected from the field.
Ethics oversight	The experiment and procedures adhered to the European and Dutch legislation on animal experimentation and were approved by the Animal Experiment Committee (DEC; AVD number 1060020197507) and performed according to the guidelines of the Leiden University Committee for Animal Experimentation.

Note that full information on the approval of the study protocol must also be provided in the manuscript.

Plants

Seed stocks	N/A
Novel plant genotypes	N/A
Authentication	N/A
